# Supplementary material for: A conformation-locking inhibitor of SLC15A4 with TASL proteostatic anti-inflammatory activity
Source: Nat Commun. 2023 Oct 20;14:6626. doi: 10.1038/s41467-023-42070-3 (PMC10589233; doi:10.1038/s41467-023-42070-3)

Supplementary Information

A conformation-locking inhibitor of SLC15A4 with TASL proteostatic anti-inflammatory activity

Andras Boeszoermenyi, Léa Bernaleau, Xudong Chen, Felix Kartnig, Min Xie, Haobo Zhang, Sensen Zhang, Maeva Delacrétaz, Anna Koren, Ann-Katrin Hopp, Vojtech Dvorak, Stefan Kubicek, Daniel Aletaha, Maojun Yang, Manuele Rebsamen*, Leonhard X Heinz*, Giulio Superti-Furga*

*Corresponding author. Email: gsuperti@cemm.oeaw.ac.at, leonhard.heinz@meduniwien.ac.at, manuele.rebsamen@unil.ch

This file contains:

- Supplementary Figure 1-11
- Supplementary Table 1-2
- Supplementary Note 1-2
- Source Data - Uncropped immunoblots

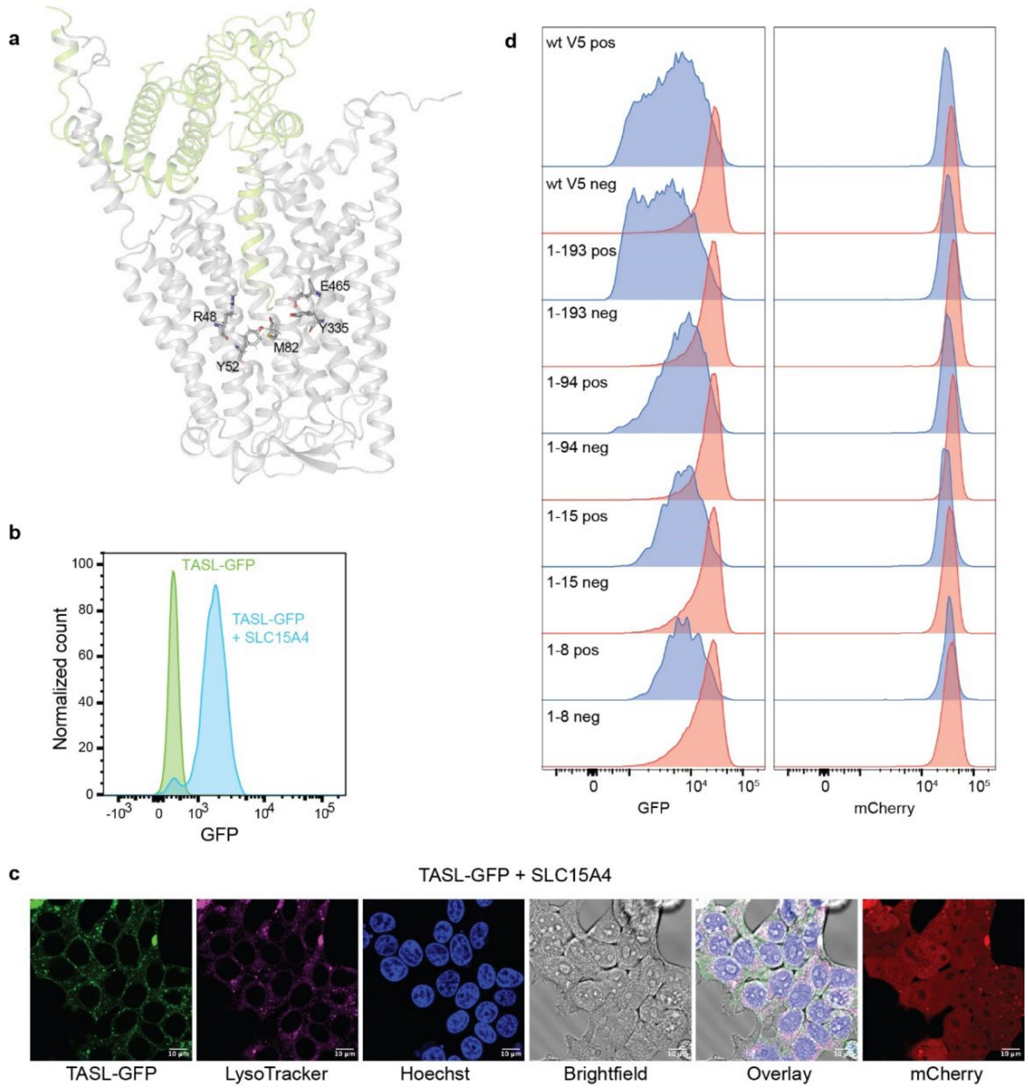

Supplementary Figure 1. SLC15A4-TASL complex controls TASL stability. (**a**) AlphaFold-predicted model of SLC15A4-TASL complex. TASL shown in green, SLC15A4 in grey. Residues positively tested for interaction in Figure 1c are shown as sticks. (**b**) Flow cytometry profiles of TASL-GFP-expressing HEK293T cells without (green) or with co-expression of SLC15A4 (blue). (**c**) Live confocal images of TGC cell line. TASL-GFP is shown in green, lysosomes were stained with lysotracker deep red (magenta), nuclei were stained with Hoechst34580 (blue), transmission (grey) and mCherry (red). Scale bars, 10 µm. (**d**) Flow cytometry analysis of TGC reporter clone transfected with doxycycline-inducible V5-tagged TASL fragments and stained 24h after induction with anti-V5 antibodies. GFP or mCherry fluorescence intensities were assessed in V5 positive and negative cells. (**b-d**) data are representative of at least two independent experiments. (**b,d**) See Supplementary Note 2 for gating strategy.

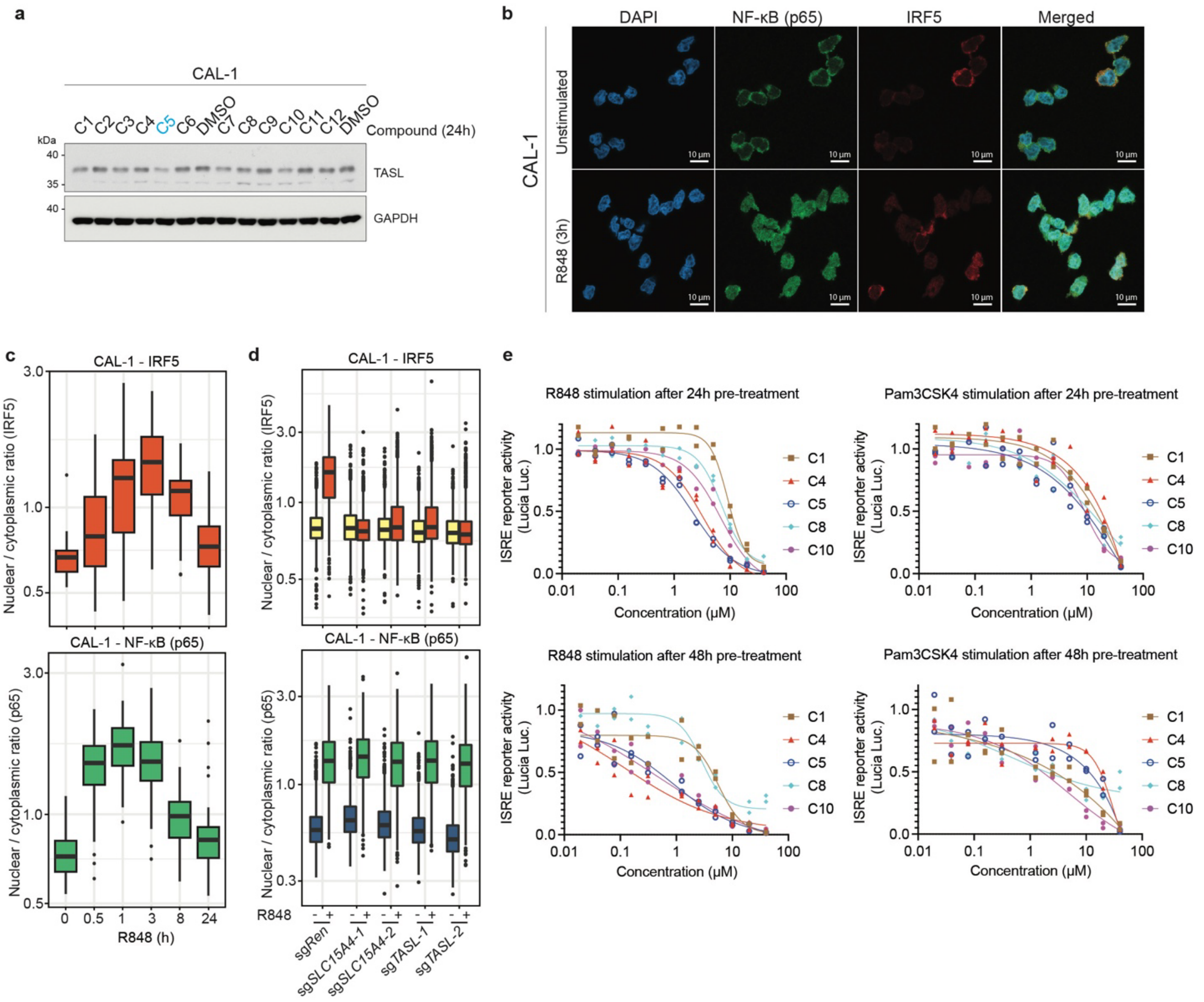

Supplementary Figure 2. Validation of HTS hits and selection of C5. (**a**) Immunoblots of CAL-1 cells treated for 24h with the indicated compounds (10 μM) or DMSO. (**b**) Representative confocal microscopy images of CAL-1 cells stimulated or not with R848 (5 μg/ml) for 3h. Scale bars, 10 μm. (**c**) Image-based evaluation of IRF5 (top) and NF-κB (bottom) nuclear translocation in CAL-1 cells. Cells were stimulated with R848 (5 μg/ml) for the indicated time and analyzed by confocal microscopy. n numbers (cells, left to right) = 19, 78, 53, 28, 27, 44. (**d**) CAL-1 control (sg*Ren*) or knockout cell lines (sg*SLC15A4*-1,2; sg*TASL*-1,2) were stimulated with R848 (5 μg/ml) for 3h as indicated and analyzed by confocal microscopy for IRF5 and NF-κB p65 nuclear translocation. n numbers (cells, left to right) = 1672, 1443, 1519, 1636, 1506, 1812, 1161, 1555, 1155, 1477. (**e**) Dose responses of five top candidates in THP1 ISRE reporter cells. Cells were pre-treated for 24h (top) and 48h (bottom) before stimulation for 20h with R848 (5 μg/ml) or Pam3CSK4 (0.1 μg/ml) as indicated. Data show n=2 biological replicates. Representative of two independent experiments. In (**a-d**) data are representative of at least two independent experiments. (**c,d**) In boxplots, bars indicate the median, boxes indicate the first to third quartiles. The top whisker extends from

hinge to largest value no further than 1.5× interquartile range (IQR) from the hinge, and the bottom whisker extends from the hinge to smallest value at most 1.5× IQR of the hinge. Dots indicate outlying points.

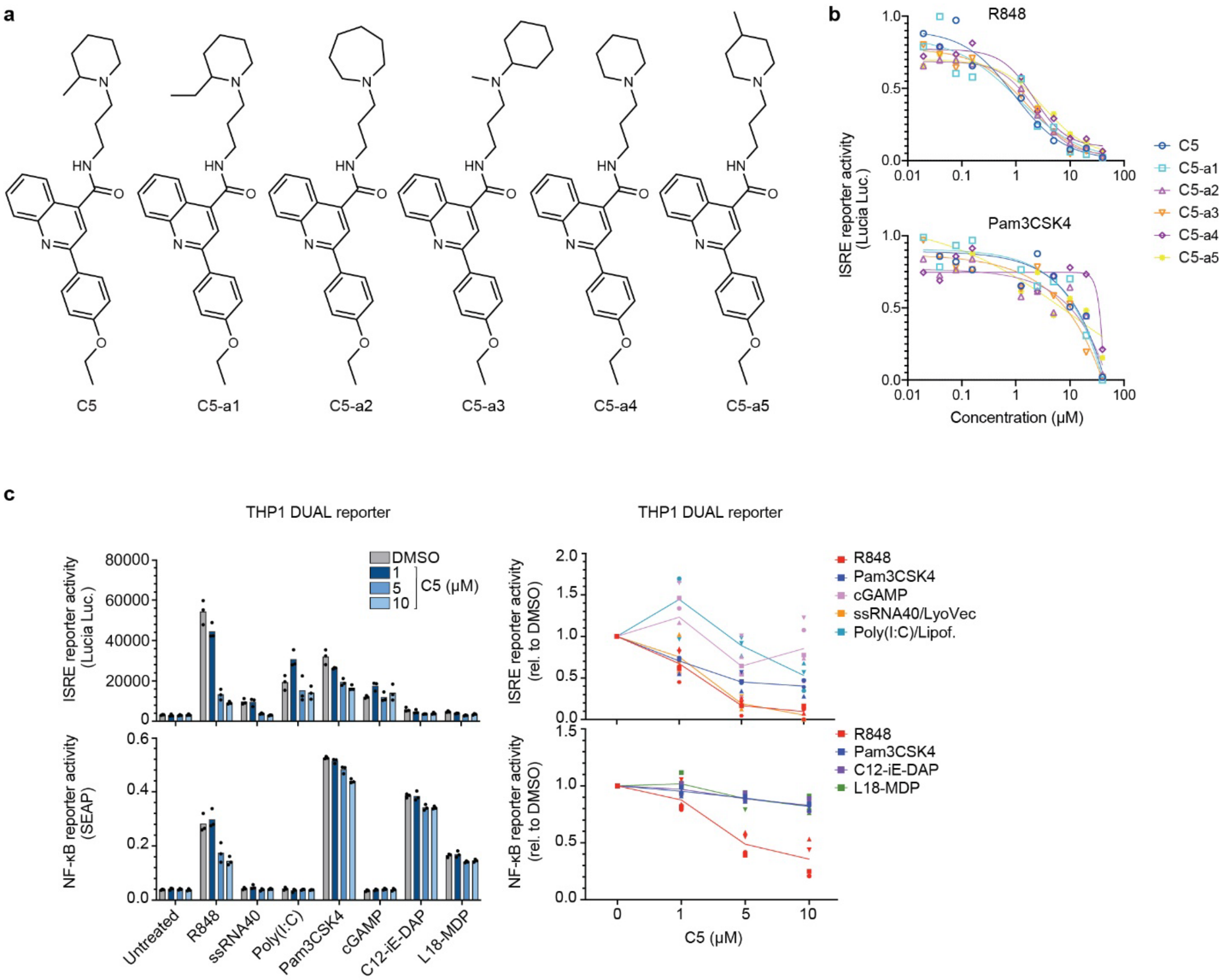

Supplementary Figure 3. Specificity of the C5 scaffold as inhibitor of TLR7/8-mediated responses. (**a**) Chemical structures of C5 and five analogs. (**b**) Dose responses of five C5 analogs in THP1 DUAL reporter cells. Cells were pre-treated for 48h before stimulation with R848 (5µg/ml) or Pam3CSK4 (0.1 µg/ml) as indicated and supernatants analyzed for ISRE reporter activity. Data from one experiment, representative of two independent experiments. (**c**) THP1 DUAL cells were pre-treated for 24h before stimulation with R848 (5 µg/ml), Pam3CSK4 (0.1 µg/ml), cGAMP (3 µg/ml), single-stranded (ss)RNA40 complexed with LyoVec (5 µg/ml), C12-iE-DAP (5 µg/ml), L18-MDP (10 µg/ml), Poly(I:C) complexed with Lipofectamine (1 µg/ml) for 24h. Supernatants were analyzed for ISRE and NF-κB reporter activity. (Left) Data show mean from one representative experiment performed in stimulation triplicates. (Right) Reporter activity relative to DMSO. Data show independent experiments (ISRE: R848 and cGAMP n=5; Pam3CSK4 n=4; ssRNA40 and Poly(I:C) n=3. NF- κB: n=4). Line represents the mean.

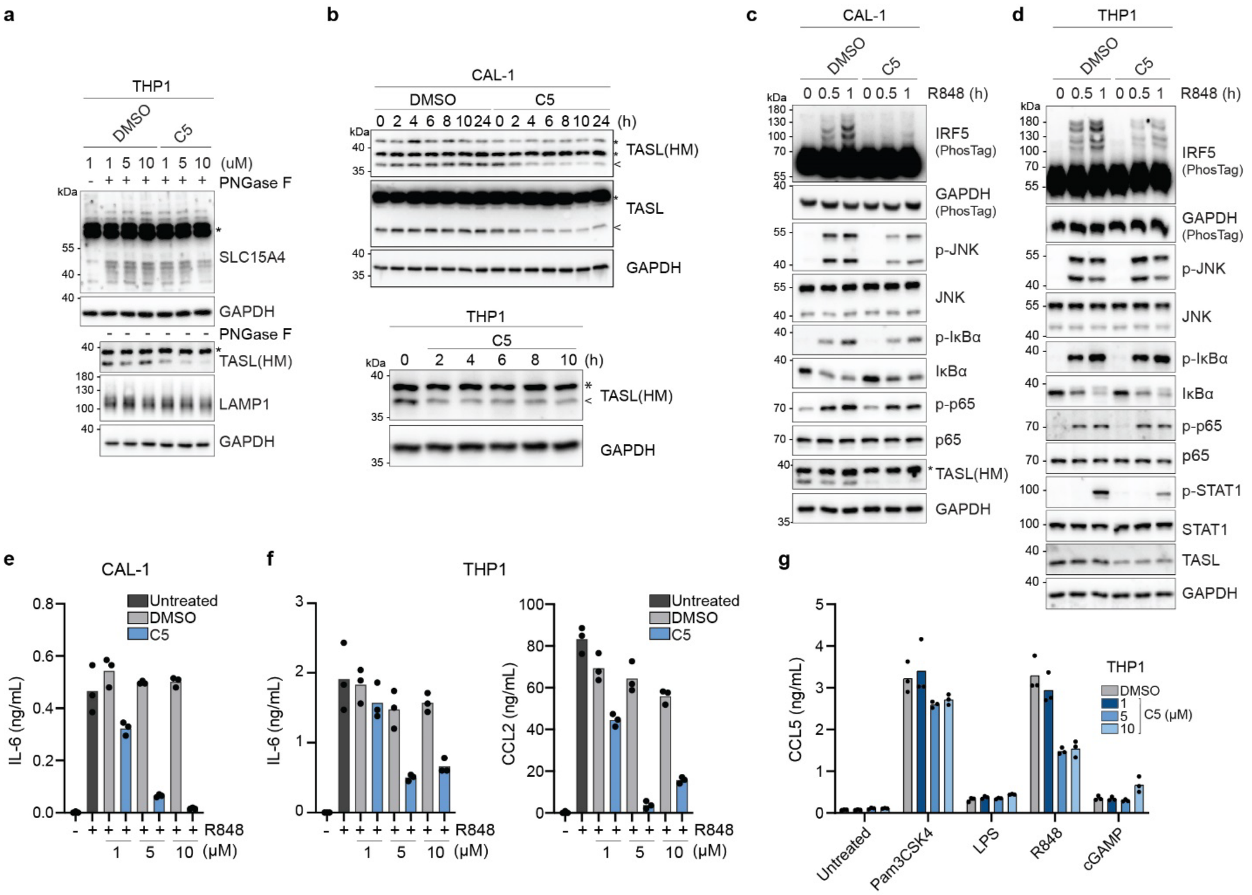

Supplementary Figure 4. C5 destabilizes TASL and impairs TLR7/8-mediated responses. (**a**) Immunoblots of THP1 upon 24h treatment with C5 or vehicle DMSO. Lysates were treated with PNGase F as indicated. (**b**) Immunoblots of CAL-1 or THP1 treated with C5 (5 µM) or DMSO for the indicated time. (**c-d**) Immunoblots of CAL-1 (**c**) or THP1 cells (**d**) pre-treated with C5 (10 µM) for 48h before R848 (5 µg/ml) treatment for the indicated time. (**e-f**) Supernatants from CAL-1 (**e**) or THP1 (**f**) cells pre-treated with C5 for 48h before R848 (5 µg/ml, 24h) stimulation were analyzed by ELISA for the indicated cytokines. Data show mean of three stimulation replicates from one experiment representative of two independent experiments. (**g**) Supernatants from THP1 cells pre-treated with C5 for 24h before stimulation (5 µg/ml R848, 0.1 µg/ml Pam3CSK4, 0.1 µg/ml LPS, 3 µg/ml cGAMP for 16h) were analyzed by ELISA for CCL5. Data show mean of three stimulation replicates from one experiment representative of two independent experiments. In (**a-d**) data are representative of at least two independent experiments.

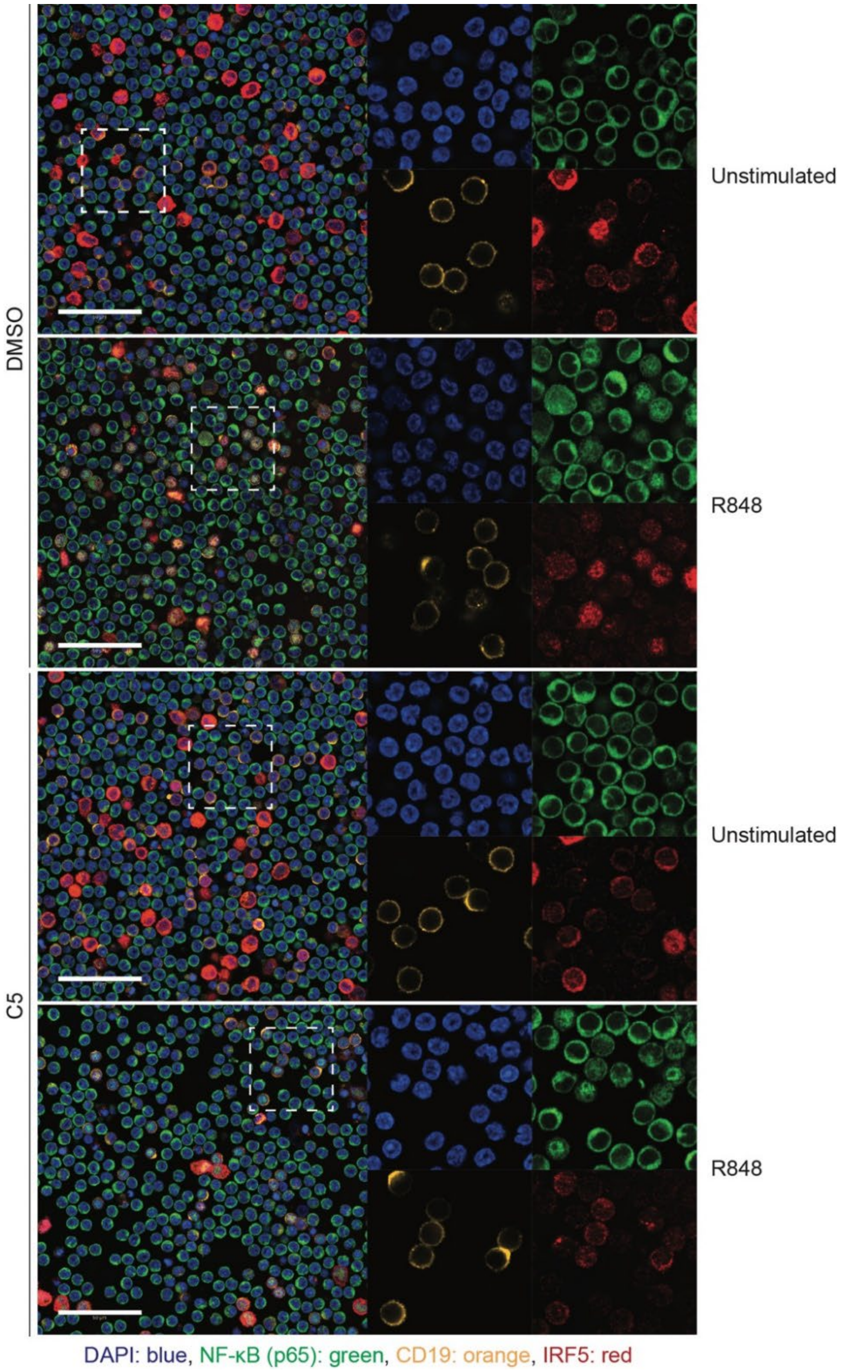

Supplementary Figure 5. Microscopy-based assessment of IRF5 and NF-κB p65 nuclear translocation in human primary B cells. Related to Figure 3c, d. Representative confocal microscopy images of PBMCs treated for 24h with DMSO or C5 (10 µM) and stimulated for 3h with R848 (5 µg/ml) as indicated. Cells were stained for DAPI (blue), NF-κB p65 (green), CD19 (orange) and IRF5 (red). Split images show individual channels of the zoomed in regions indicated by the white squares. Scale bar: 50 µm.

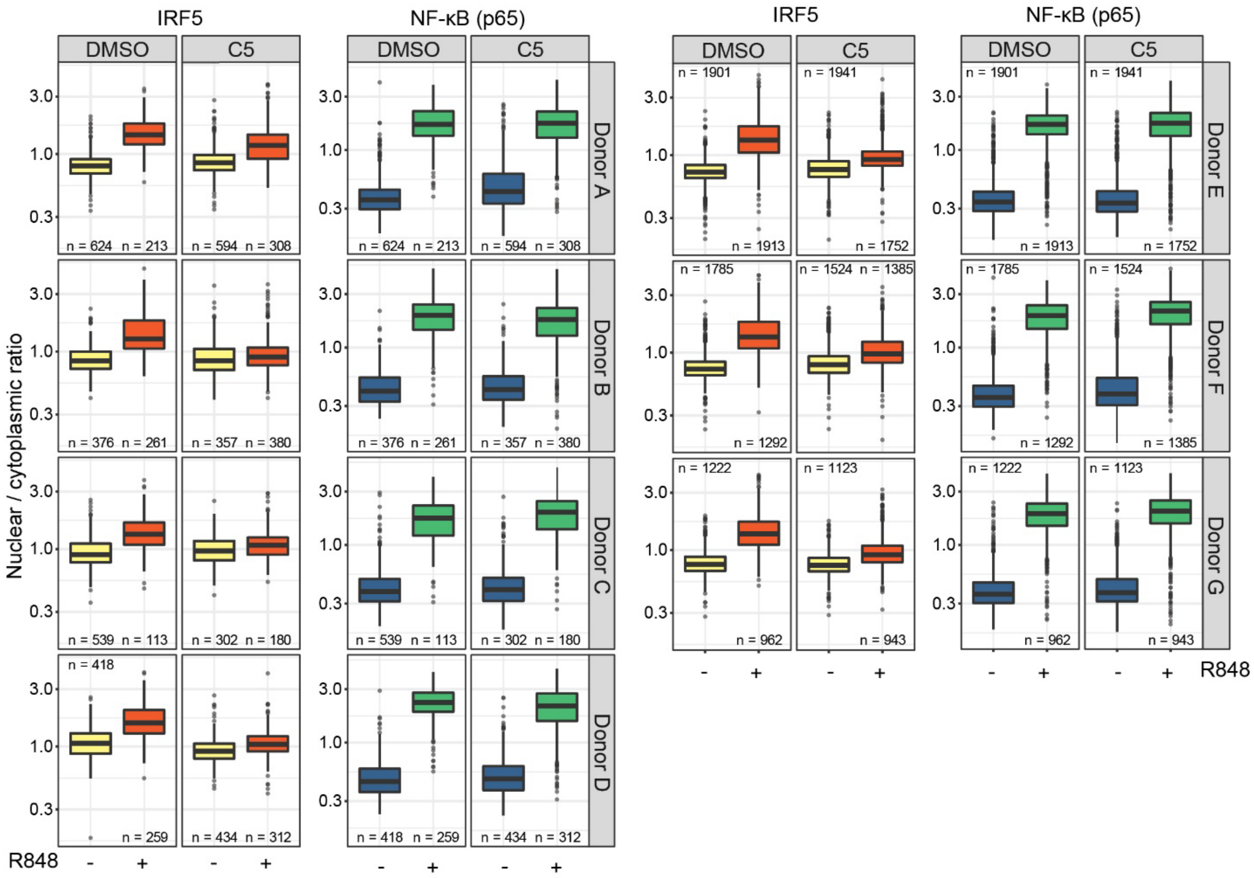

Supplementary Figure 6. Quantification of IRF5 and NF-κB p65 nuclear translocation in B cells of individual donors. Related to Figure 3d. B cells from PBMCs of 7 individual donors (A-G) pre-treated for 24h with DMSO or C5 (10 µM) and stimulated for 3h with R848 (5 µg/ml) as indicated were analyzed for IRF5 and NF-κB p65 nuclear translocation by confocal microscopy. In boxplots, bars indicate the median, boxes indicate the first to third quartiles. The top whisker extends from hinge to largest value no further than 1.5× interquartile range (IQR) from the hinge, and the bottom whisker extends from the hinge to smallest value at most 1.5× IQR of the hinge. Dots show outlying points, n (cells) as indicated.

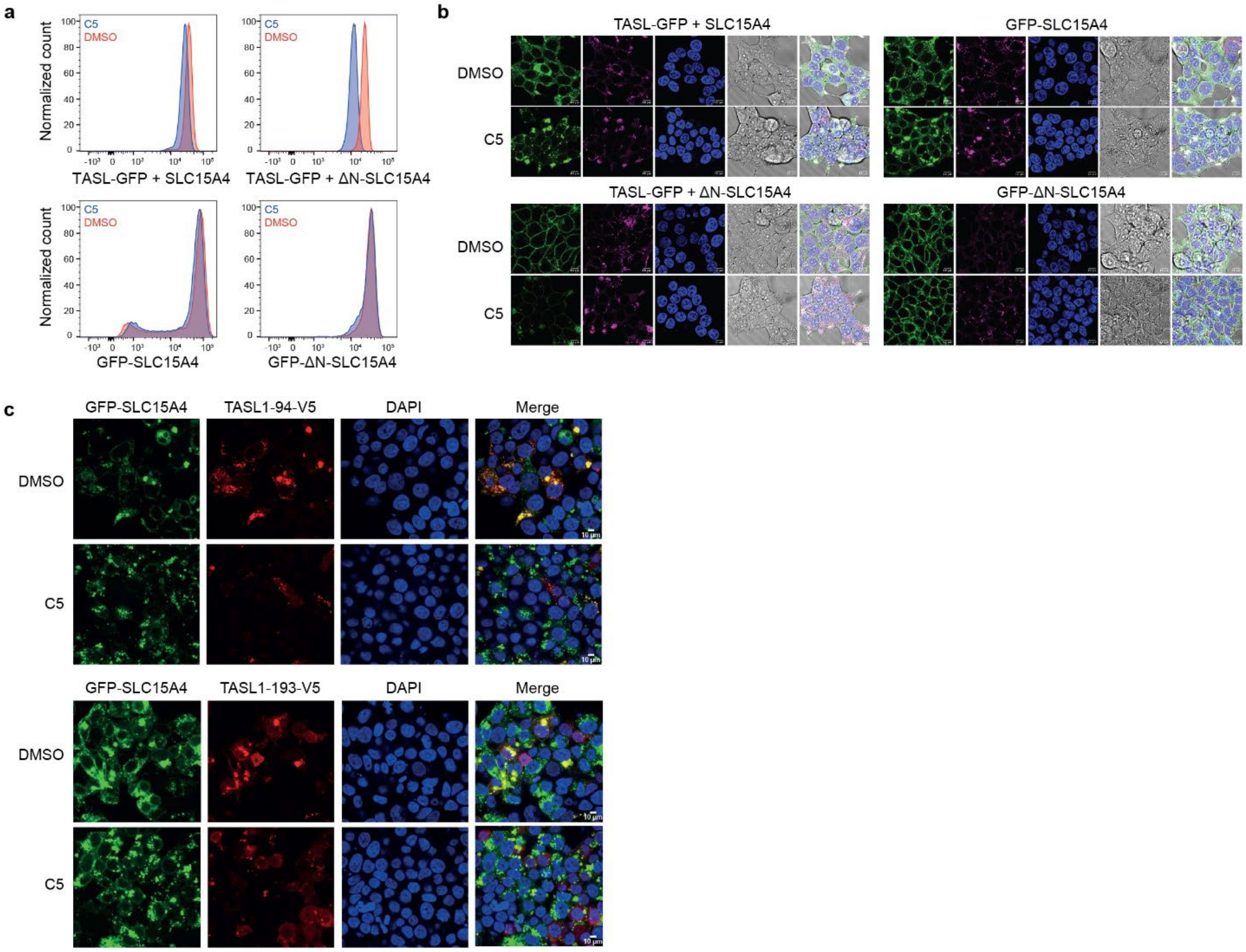

Supplementary Figure 7. C5 selectively induces TASL, but not SLC15A4 degradation. (**a, b**) Related to Figure 3g, h. Flow cytometry analysis (**a**) or live cell fluorescence microscopy images (**b**) of HEK293T cells stably expressing the indicated constructs after 24h treatment with DMSO or 10 µM C5. GFP (green), lysotracker (magenta), Hoechst (blue) transmission (grey) and mCherry (red). (**c**) V5-tagged TASL fragments TASL1-94-V5 and TASL1-193-V5 transiently expressed in a constitutively GFP-SLC15A4 expressing cell line were stained for V5 after 24h treatment with DMSO and 10 µM C5, respectively. (**a**) See Supplementary Note 2 for gating strategy. (**a-c**) data are representative of at least two independent experiments. (**b,c**) Scale bars, 10 µm.

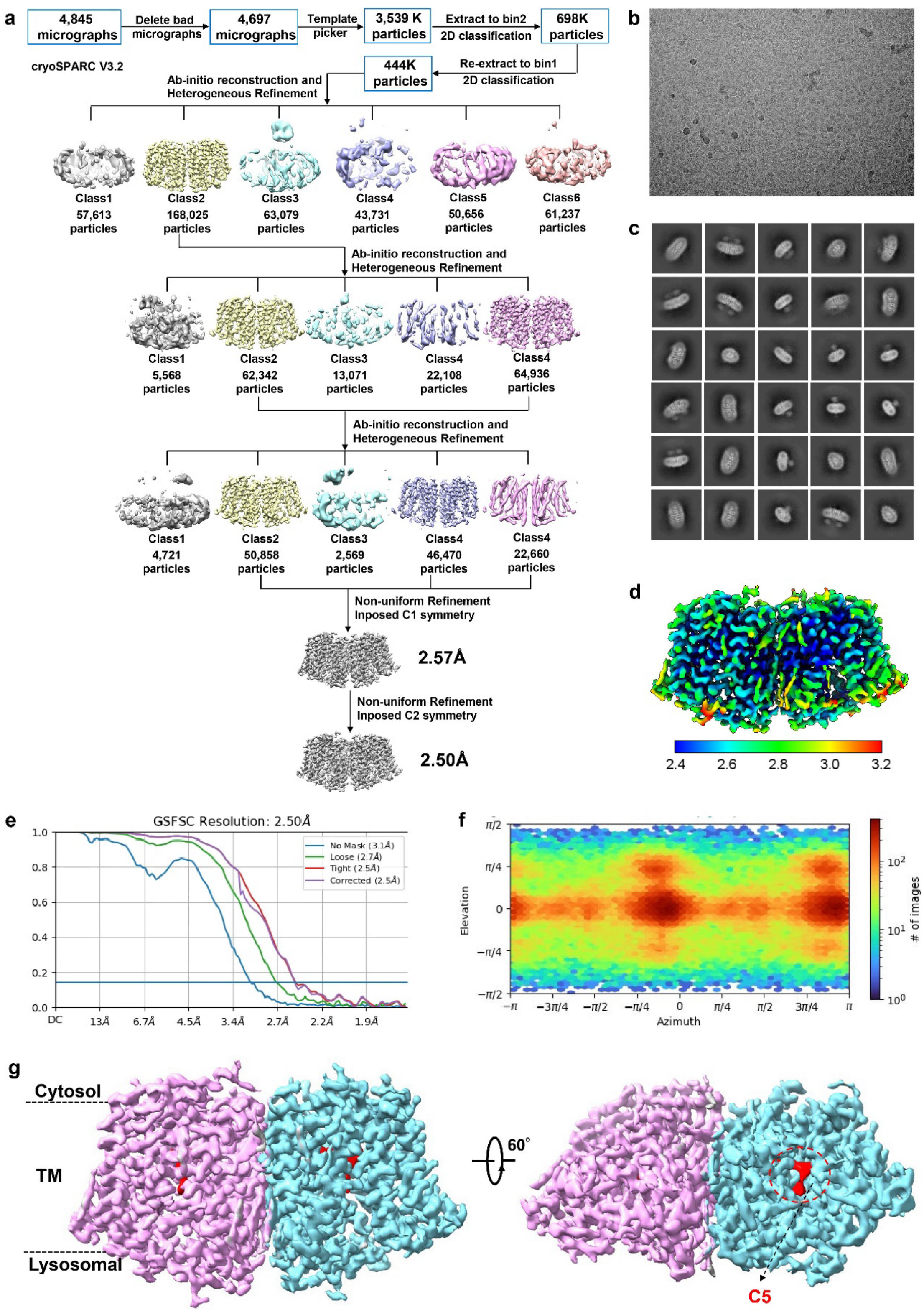

Supplementary Figure 8. Reconstruction and structure determination of human SLC15A4/C5 complex. (**a**) Human SLC15A4/C5 complex cryo-EM data processing. 444 k particles were kept after 2D classification and subjected to multiple rounds of ab-initio reconstruction and heterogenous refinement. A final dataset containing 120 k particles was used for Non-uniform and CTF refinement. (**b**) Representative

cryo-EM micrograph of human SLC15A4/C5 complex. (**c**) Representative 2D averages of human SLC15A4/C5 complex. (**d**) Local resolution map of the final 3D density map. (**e**) Gold-standard Fourier Shell correlation (FSC) curve of SLC15A4/C5 complex after 3D refinement. The resolution estimation was based on the criterion of FSC 0.143 cutoff. (**f**) Particle orientation distributions in the last iteration of the structural refinement. (**g**) 2.5-Å-resolution cryo-EM map of human SLC15A4/C5 complex. The two protomers of SLC15A4 are colored pink and cyan, respectively. The bound C5 molecules are colored red.

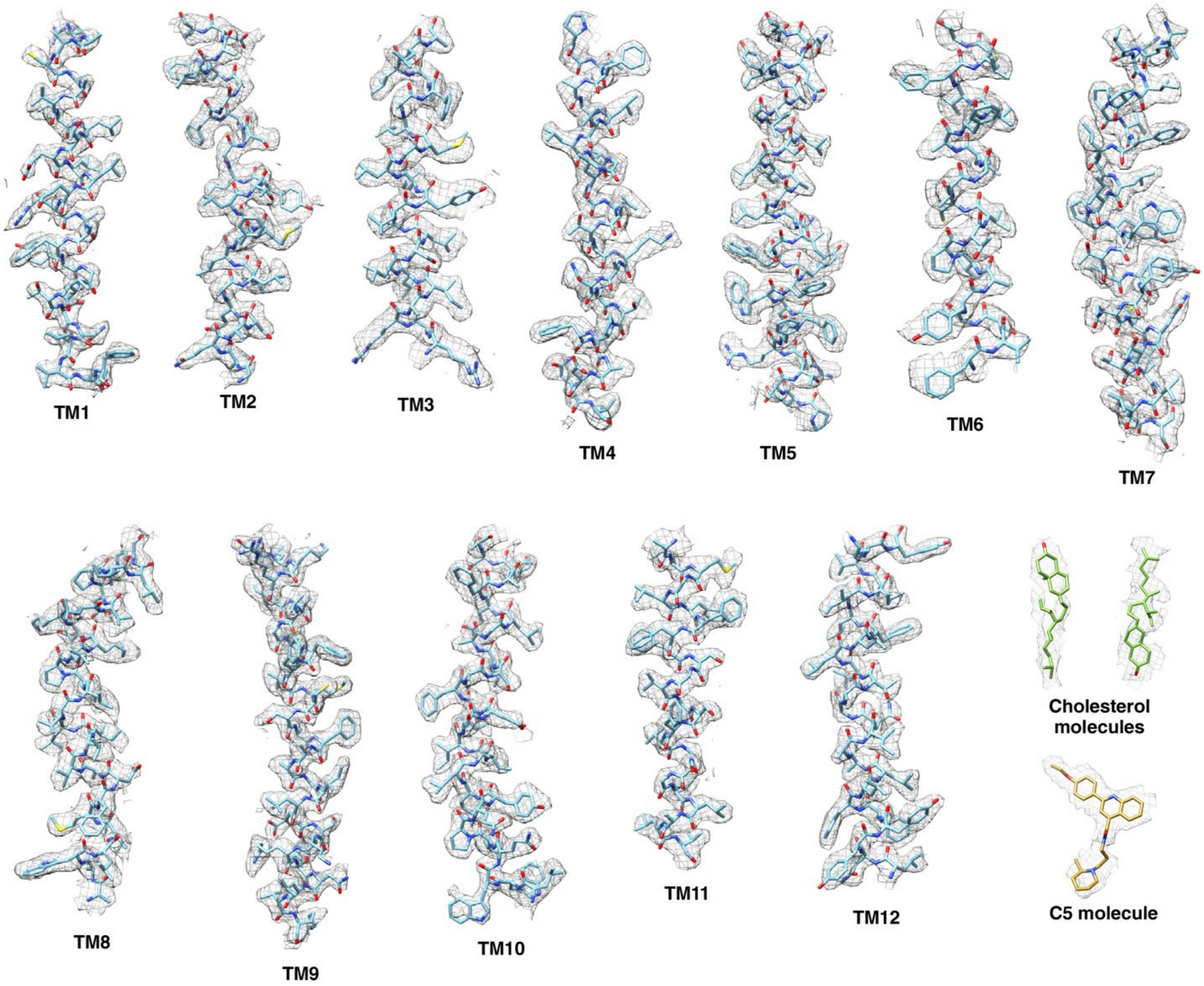

Supplementary Figure 9. Density maps of the 2.5 Å cryo-EM structure of human SLC15A4/C5 complex. Density maps of the transmembrane regions of SLC15A4, the bound cholesterol and C5 molecules. Stick style atomic models of SLC15A4 (blue), cholesterol molecules (green) and C5 molecule (yellow) were fitted into the cryo-EM density maps (gray mesh). The density maps were contoured at 6σ.

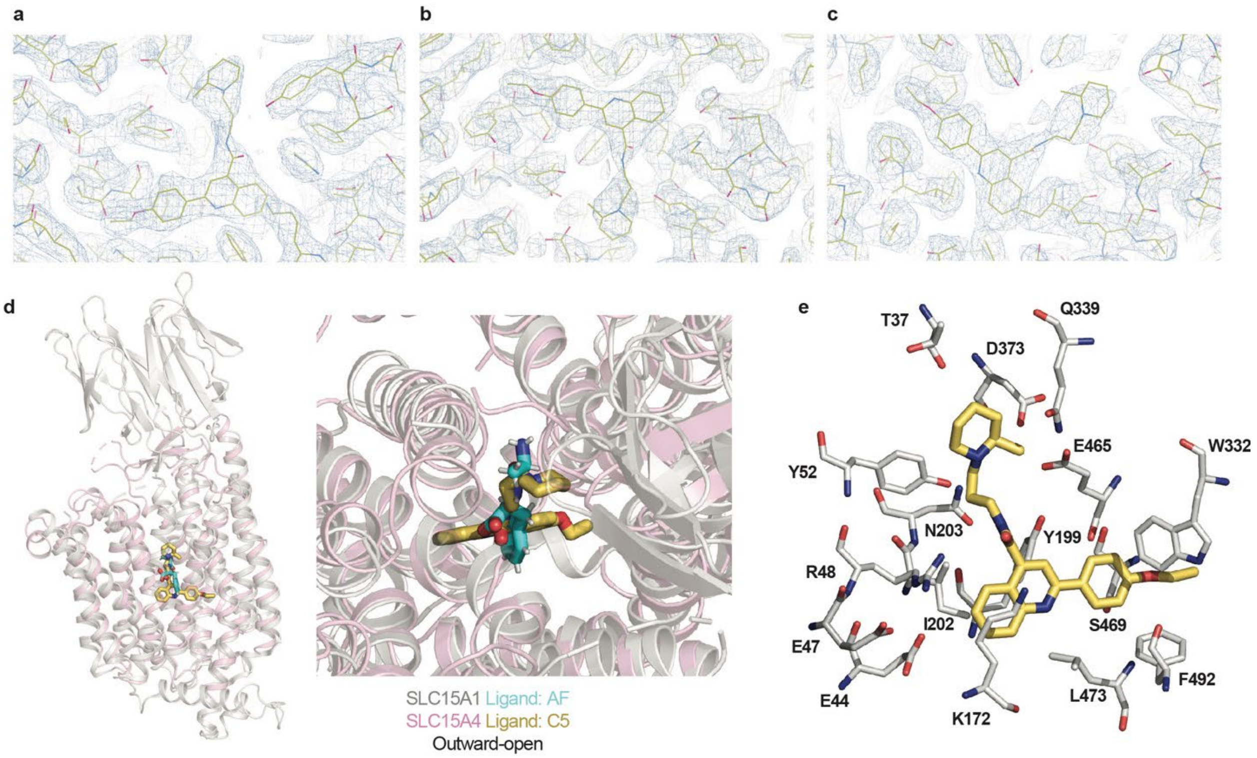

Supplementary Figure 10. C5 binds to the canonical binding pocket of POT family transporters. (**a-c**) Cryo-EM maps (contoured at 6σ) of the SLC15A4 C5 binding site with additional electron density for C5 in the center. (**d**) Structures of outward-open human SLC15A1 (PDB ID: 7PMW [https://doi.org/10.2210/pdb7pmw/pdb]) and human SLC15A4 are shown with their ligands bound, Ala-Phe (AF) dipeptide or C5 respectively, from side and top views. (**e**) Hydrophobic and charged residues surrounding C5 are shown, with C5 depicted as dark yellow sticks.

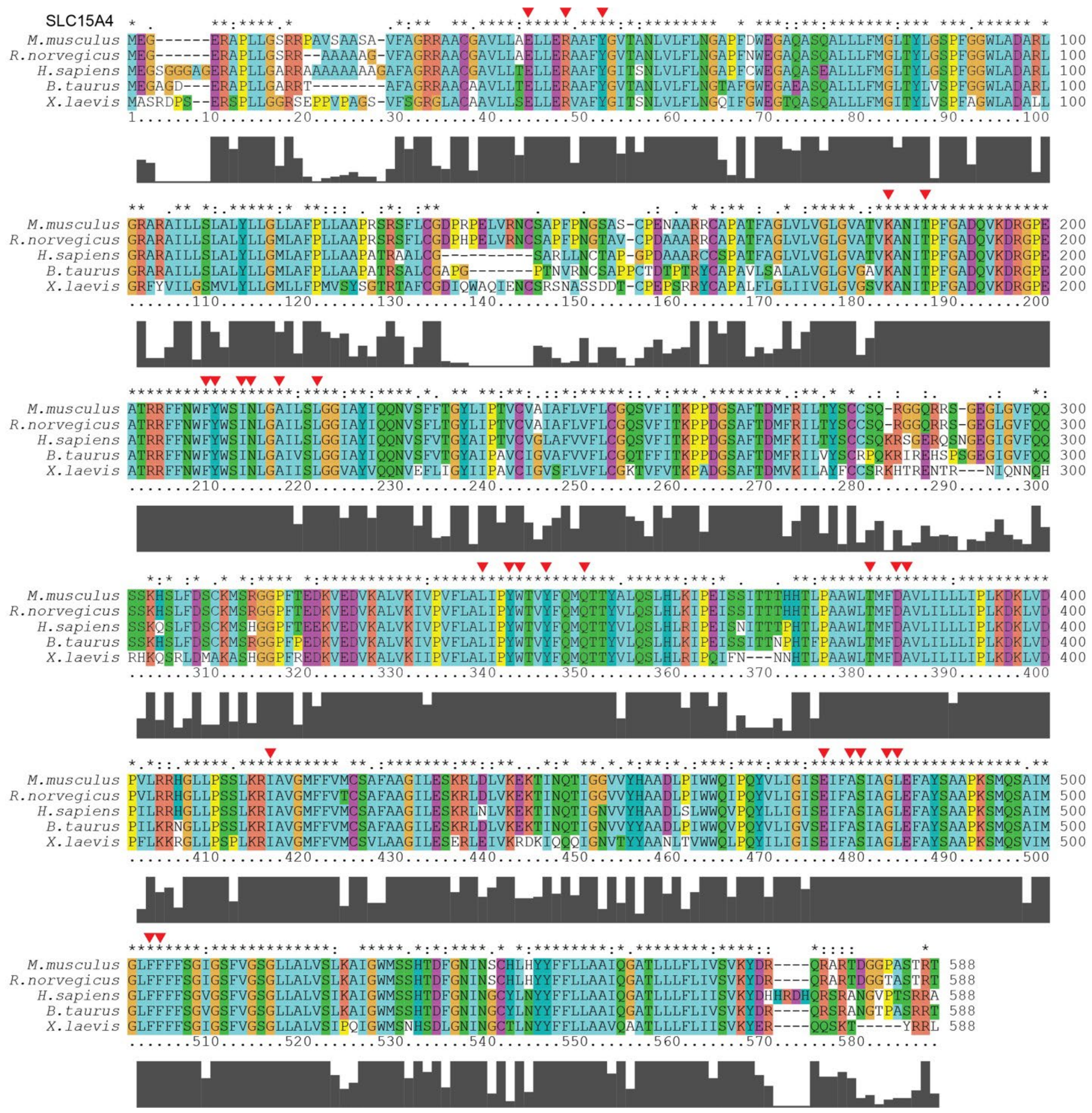

Supplementary Figure 11. C5-interacting residues are conserved among vertebrates. Multiple sequence alignment of SLC15A4 protein from representative vertebrate species. UniProt entry names: S15A4_MOUSE, S15A4_RAT, S15A4_HUMAN, S15A4_BOVIN, S15A4_XENLA. Red triangles above alignment indicate residues surrounding C5, related to Figure 4f and Figure S10e.

Supplementary Table 1. Small molecule screening data

Category	Parameter	Description
Assay	Type of assay	Phenotypic assay
	Target	SLC15A4 – TASL interaction
	Primary measurement	Fluorescence of TASL-GFP reporter
	Key reagents	Stable cell TGC cell line
	Assay protocol	Pre-plate compounds at 10µM. Add 2k cells in 50µL medium in 384-well plate format. Incubate for 40-50h. Measure GFP and mCherry fluorescence
	Additional comments	Co-expressed mCherry was used for normalization
Library	Library size	86.727
	Library composition	The compound library consisted of a large structural diversity collection, and subcollections of annotated compounds. This includes the NIH clinical collection, natural products, approved drugs, known bioactives (e.g. kinase, epigenetic modifiers, ...), and other drug-like molecules.
	Source	CeMM – Molecular Discovery Platform
	Additional comments	
Screen	Format	384-well plate
	Concentration(s) tested	10µM
	Plate controls	DMSO
	Reagent/ compound dispensing system	Compounds were dispensed with the acoustic LabCyte Echo 550 liquid handler. Cells were dispensed with a Thermo Multidrop Combi dispenser.
	Detection instrument and software	Operetta CLS High Content Analysis system at one field per well and 20x magnification
	Assay validation/QC	Initial assay validation was done with siRNAs targeted towards SLC15A4 and a pre-screen with 2k compounds
	Correction factors	Cell number, cell roundness
	Normalization	mCherry fluorescence
	Additional comments	
Post-HTS analysis	Hit criteria	Reduction of GFP/mCherry ratio > 15% and GFP > 5% and visual inspection
	Hit rate	0.014% (12 out of 86.727)
	Additional assay(s)	THP1 DUAL assay, IRF5 translocation assay
	Confirmation of hit purity and structure	Resynthesis by several vendors
	Additional comments	Identity and purity of the hit compound C5 was confirmed by MS and NMR

Supplementary Table 2: Cryo-EM data collection, refinement and validation statistics

	SLC15A4-C5 complex
**Data collection and processing**	
Magnification	105,000
Voltage (kV)	300
Electron exposure (e^{-}/Å^{2})	50
Defocus range (μm)	-1.0 ~ -2.5
Pixel size (Å)	0.8433
Software	cryoSPARC
Symmetry imposed	C2
Initial particle images (no.)	3,539,726
Final particles images (no.)	119,988
Map resolution (Å)	2.50
FSC threshold	0.143
Local map resolution range (Å)	2.4-3.2
**Refinement**	
Software	PHENIX1.14
Model resolution (Å)	2.50/2.80
FSC threshold	0.143/0.5
Map sharpening *B* factor	84.8
Model composition	
Non-hydrogen atoms	7108
Protein residues	910
Ligand	6
B factors (Å^{2})	
Protein	50.49
Ligand	33.78
R.m.s deviations	
Bond length (Å)	0.007
Bond angles (°)	1.293
Validation	
MolProbity score	1.94
Clashscore	10.07
Poor rotamers (%)	0.28
Ramachandran plot	
Favored (%)	93.71
Allowed (%)	6.07
Disallowed (%)	0.22

Supplementary Note 1:

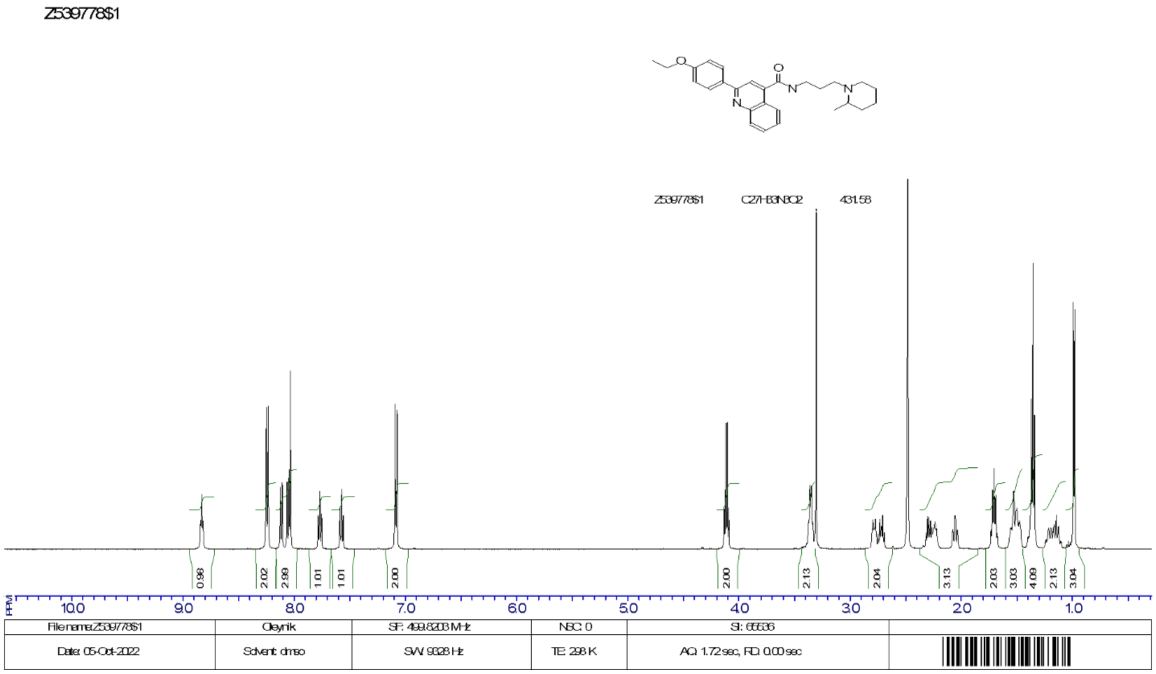

^{1}H-NMR spectrum of C5 as provided by Enamine.

MaxPeak: 100.00%
Ret_Time: 1.156 min

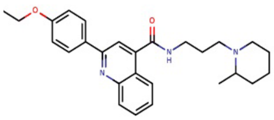

Mol Wt 431.57
Exact Mass 431.31

#	Time	Area%
1	1.156	100.00

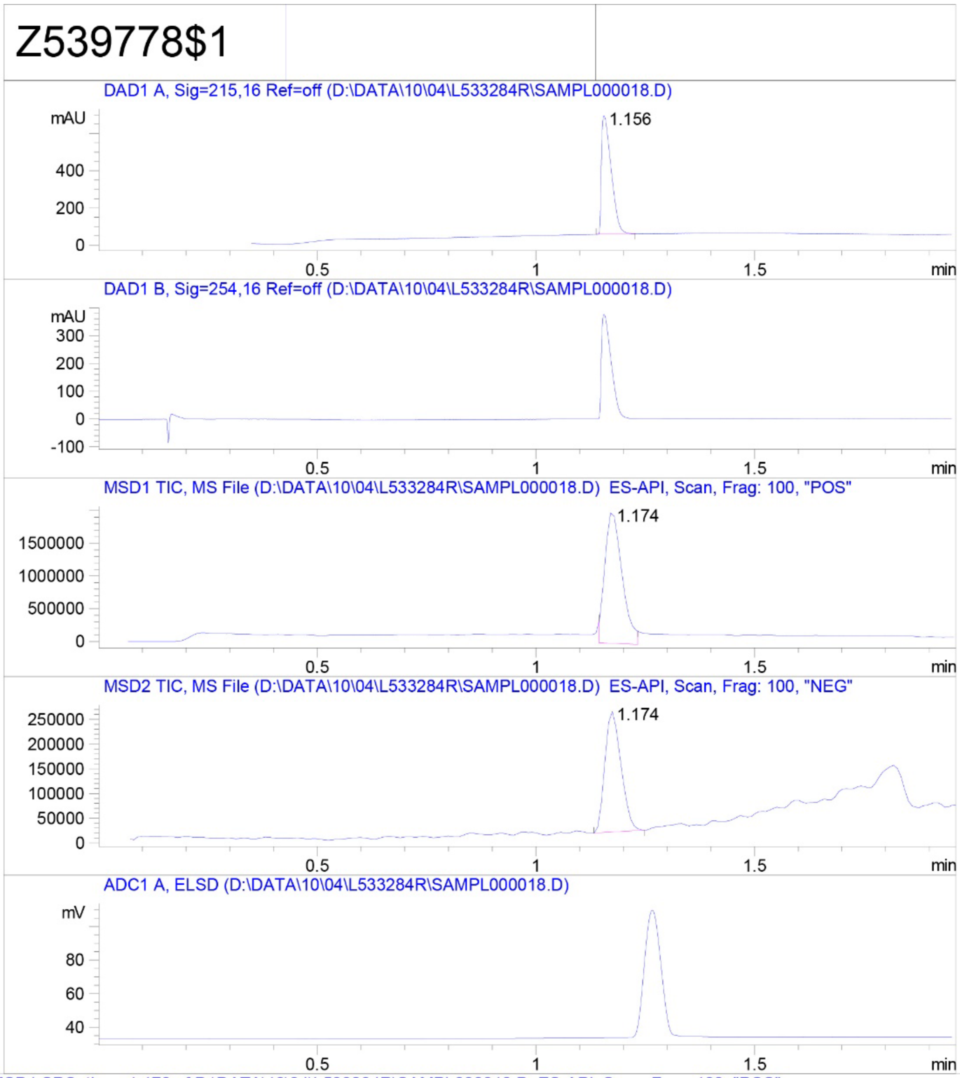

RT 1.174

RT 1.174

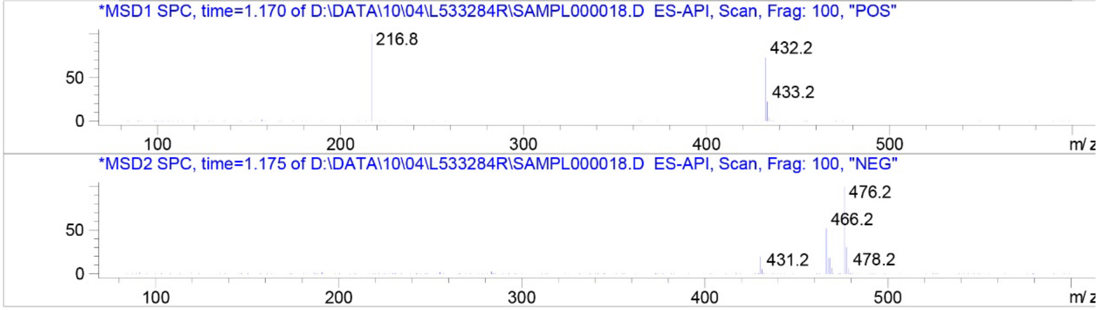

HPLC chromatograms and MS traces for C5 as provided by Enamine.

Supplementary Note 2

Gating strategy for Figure S1b:

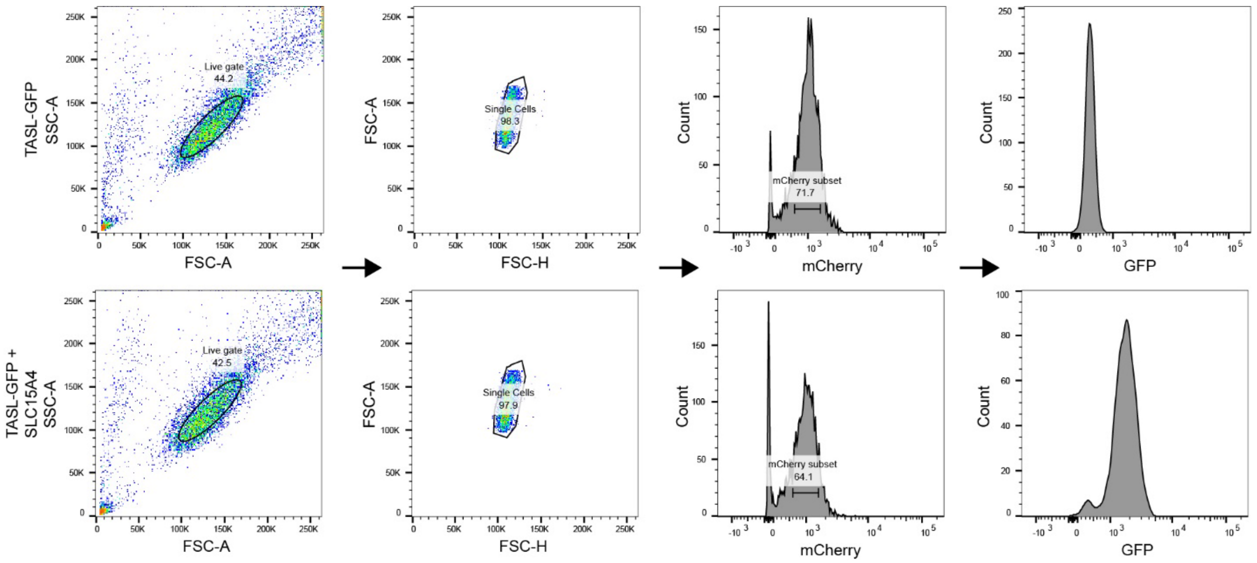

Gating strategy to determine the effect of SLC15A4 co-expression on TASL-GFP fluorescence in TGC reporter cells. Live cells are separated from debris by gating on an SSC-A vs. FSC-A plot followed by an FSC-H vs. FSC-A plot to select for single cells. A histogram of the single cells is used to extract an mCherry-expressing population that is then further analyzed for TASL-GFP fluorescence.

Gating strategy for Figure S1d

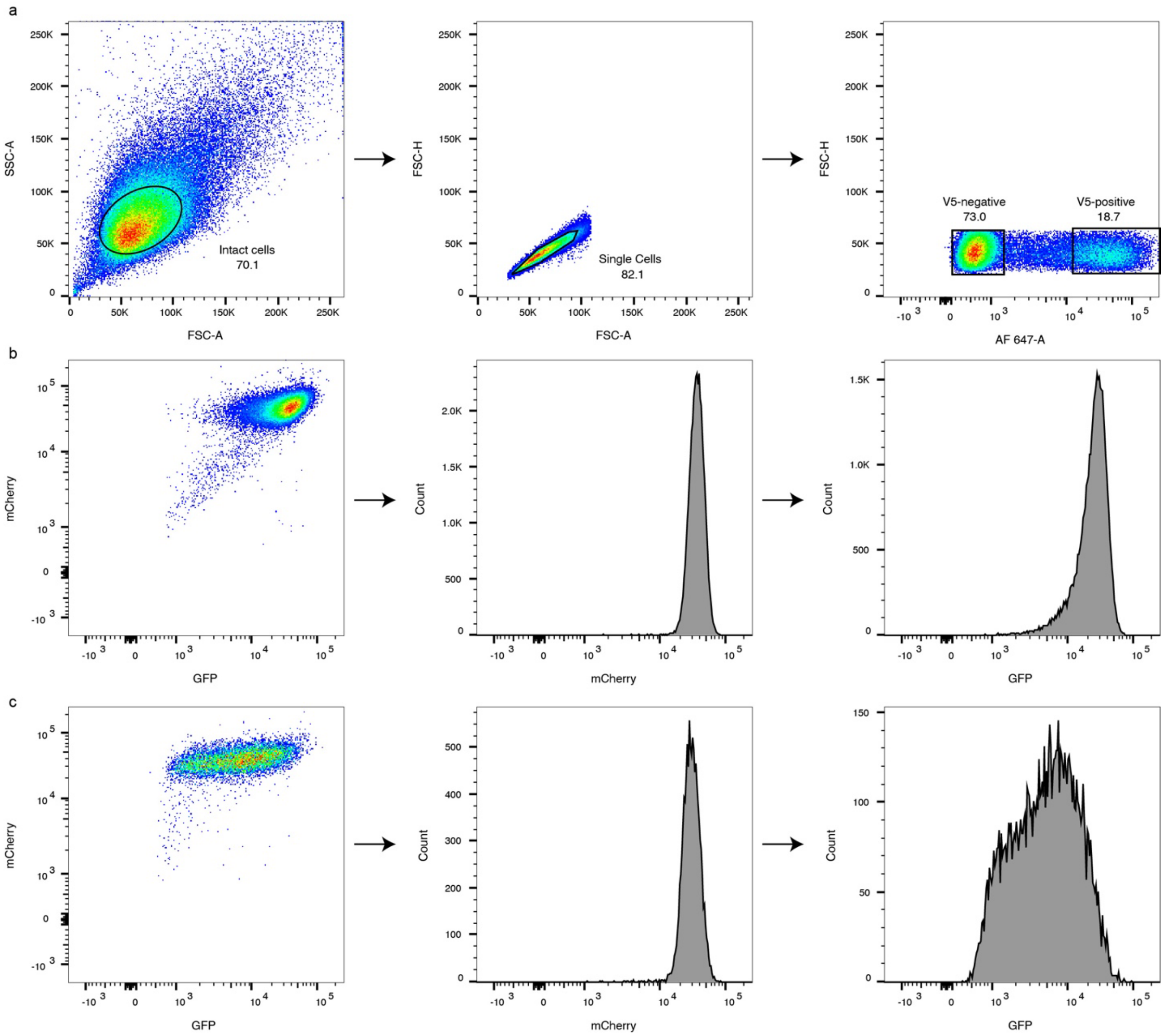

Gating strategy to quantify the effect of V5-labelled TASL on TASL-GFP fluorescence in TGC reporter cells co-expressing SLC15A4. (**a**) Live cells are separated from debris by gating on an SSC-A vs. FSC-A plot followed by a FSC-H vs. FSC-A plot used to select single cells. Next, an FSC-H vs. AF-647-A plot is applied to select for the presence of V5-labelled TASL. (**b,c**) The distribution of mCherry and GFP fluorescence in these two populations is plotted for (**b**) V5-negative (**c**) and V5-positive cells, respectively. Histograms representing the mCherry and GFP distributions are shown next to the respective scatter plots.

Gating strategy for Figure 3g and Figure S7a (TASL-GFP):

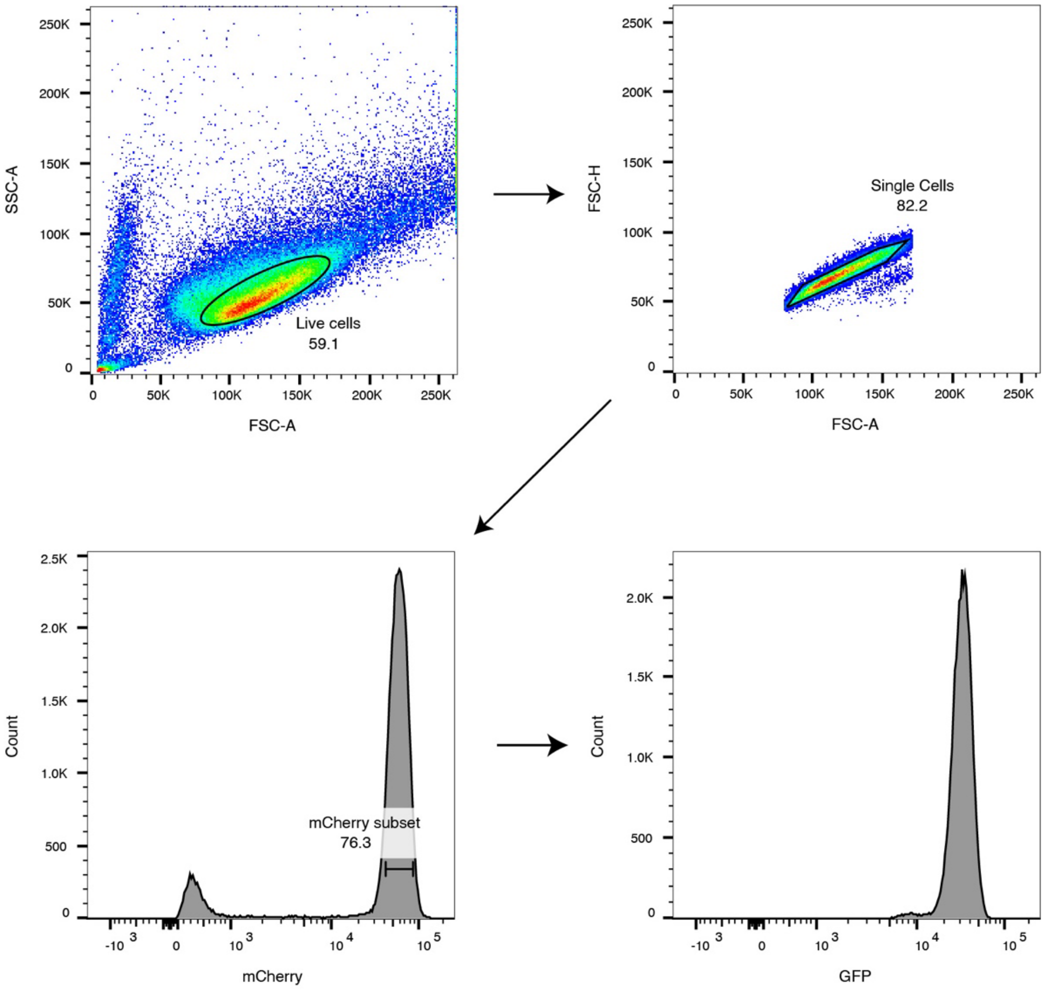

Gating strategy to quantify the effect of C5 on TASL-GFP fluorescence in TGC reporter cells in presence of SLC15A4. Live cells are separated from debris by gating on an SSC-A vs. FSC-A plot followed by an FSC-H vs. FSC-A plot to select for single cells. A histogram of the single cells is used to extract an mCherry-expressing population that is then further analyzed for TASL-GFP fluorescence. The mean GFP signal from the mCherry-subset population was extracted for compound treated and DMSO treated conditions and normalized against the value measured for the DMSO treated group for quantification.

Gating Strategy for Figure 3g and Figure S7a (GFP-SLC15A4):

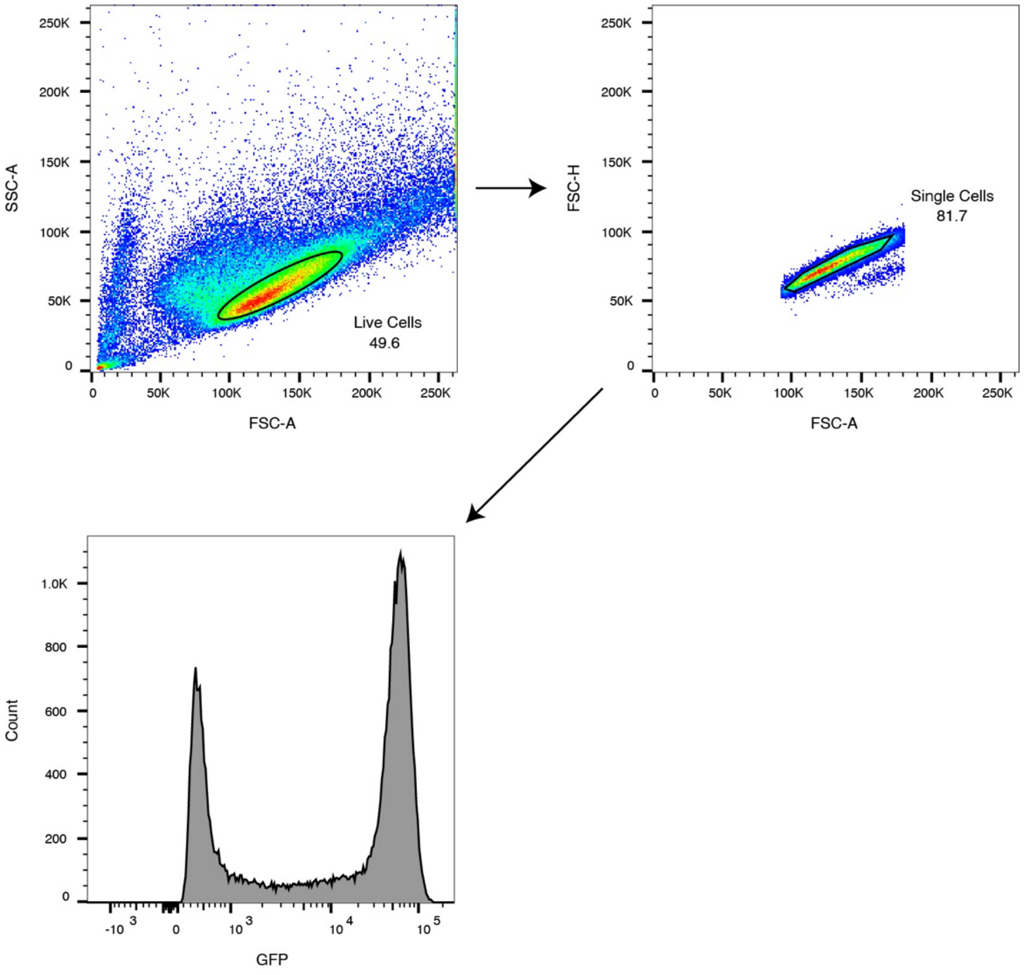

Gating strategy to quantify the effect of C5 on GFP-SLC15A4 fluorescence. Living cells are separated from debris with an SSC-A vs. FSC-A plot followed by an FSC-H vs. FSC-A plot to select for single cells. A histogram of the GFP-SLC15A4 expressing population is directly extracted from the single cell selection plot. The mean GFP signal from the single cell population was extracted for compound treated and DMSO treated conditions and normalized against the value measured for the DMSO treated group for quantification.

Source Data, Figure 1A

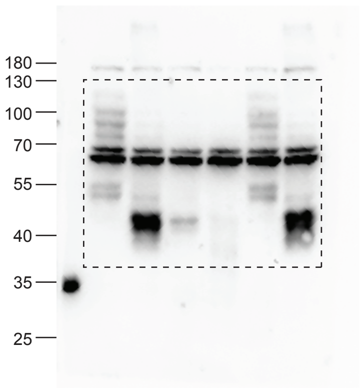

anti-SLC15A4

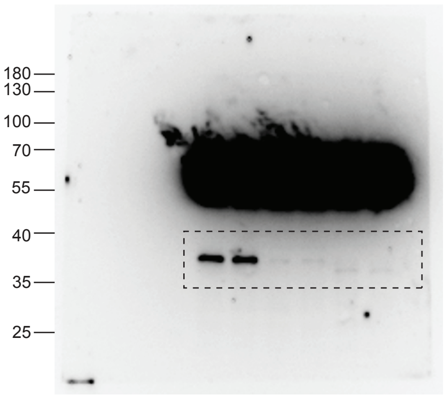

anti-TASL

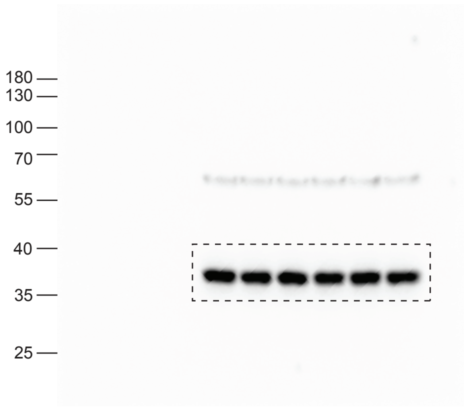

anti-GAPDH

Source Data, Figure 1C

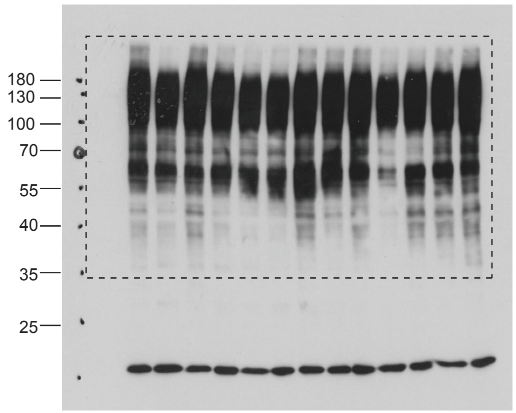

WCE, anti-HA

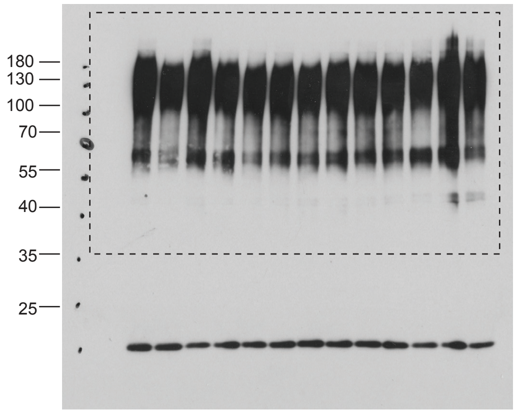

IP, anti-HA

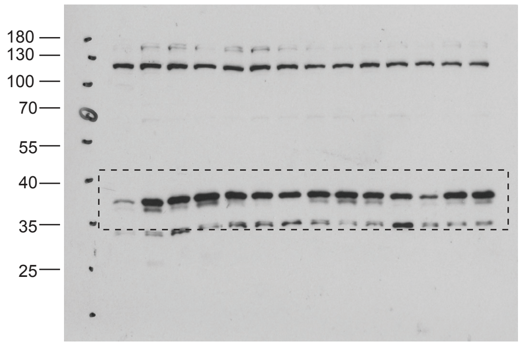

WCE, anti-TASL

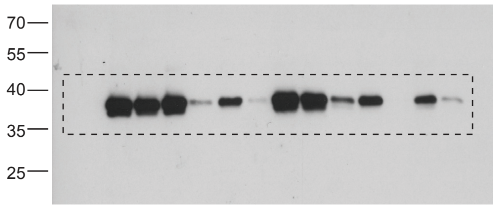

IP, anti-TASL

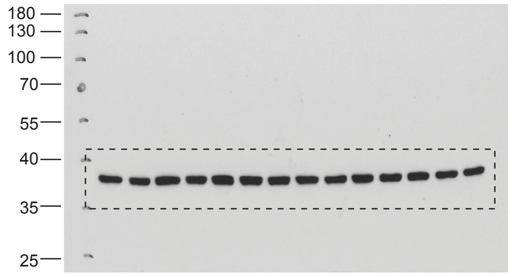

WCE, GAPDH

Source Data, Figure 1D

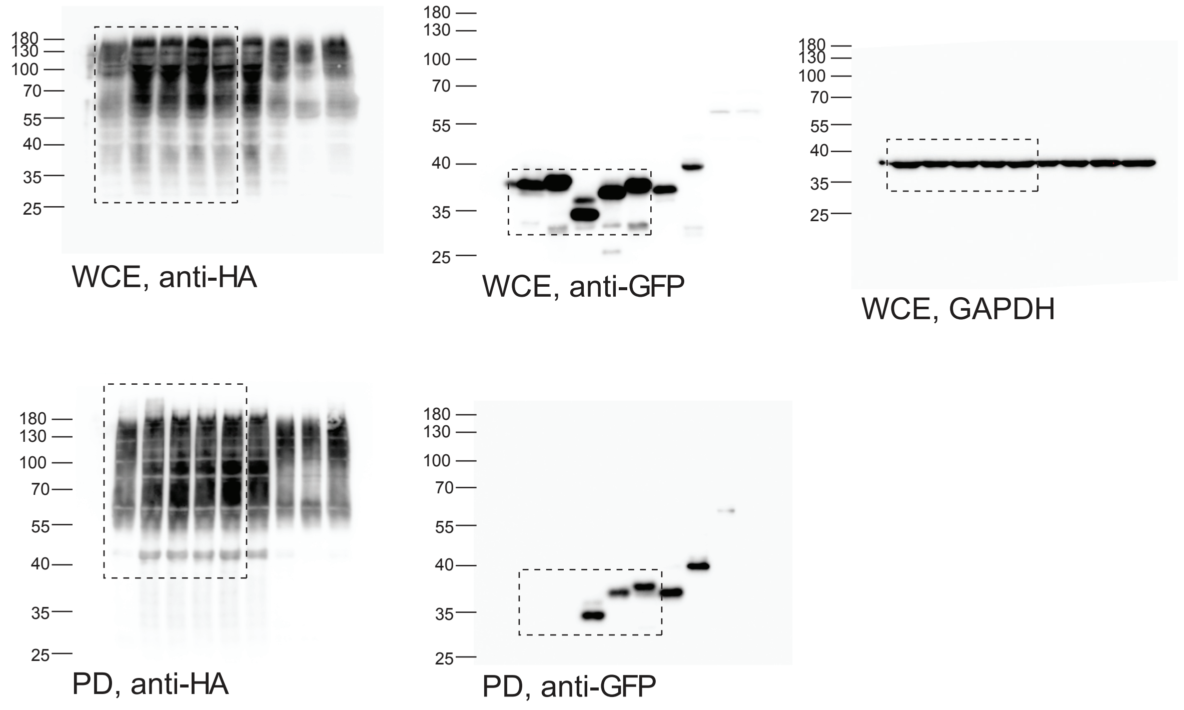

Source Data, Figure 2D

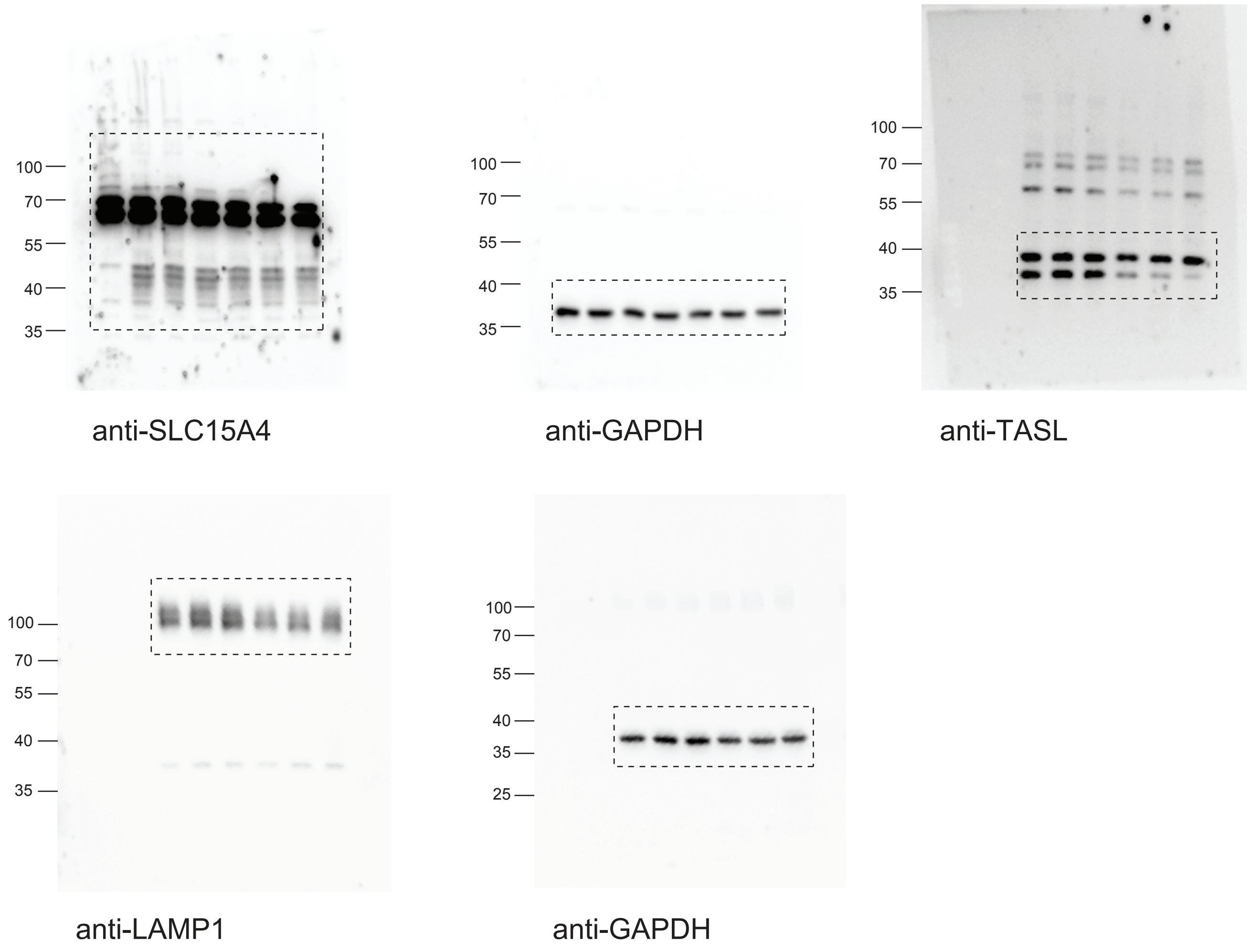

Source Data, Figure 2E

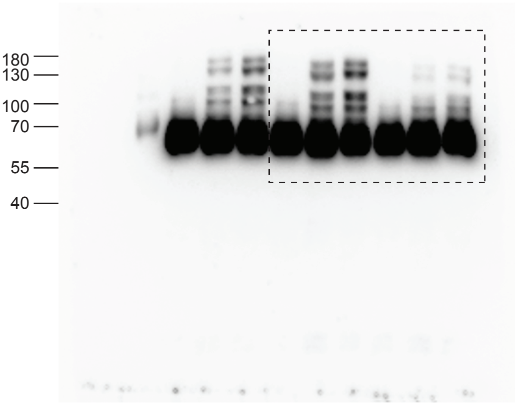

anti-IRF5 (PhosTag)

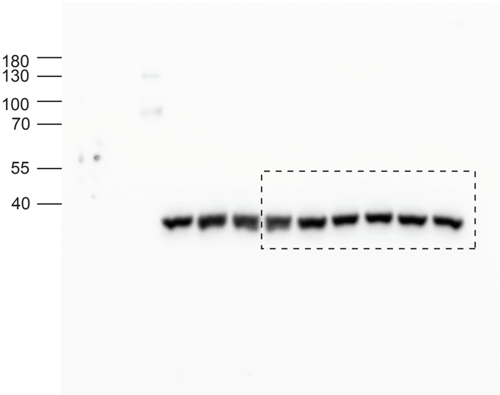

anti-GAPDH (PhosTag)

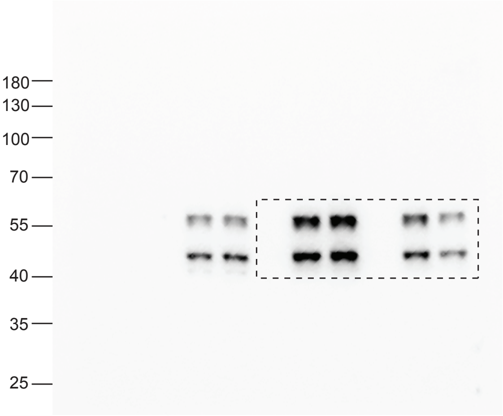

anti-p-JNK

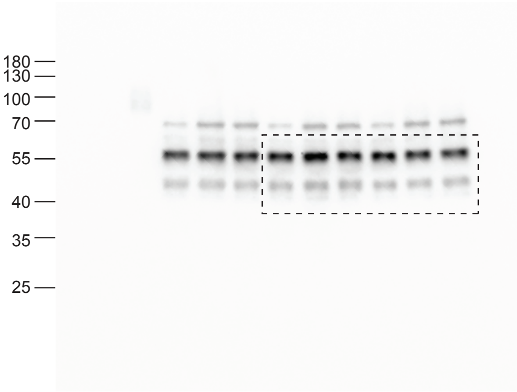

anti-JNK

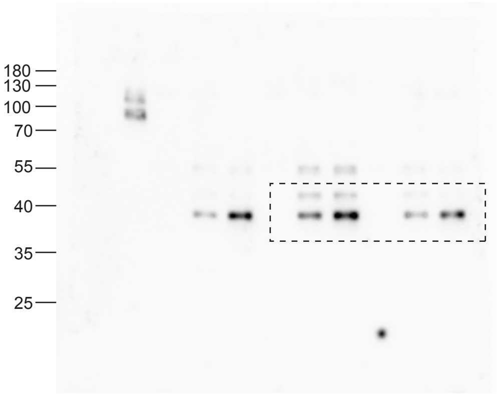

anti-p-IκBα

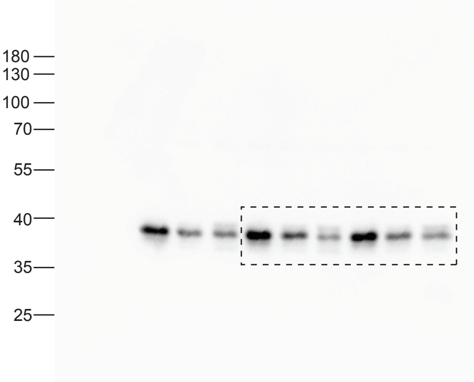

anti-IκBα

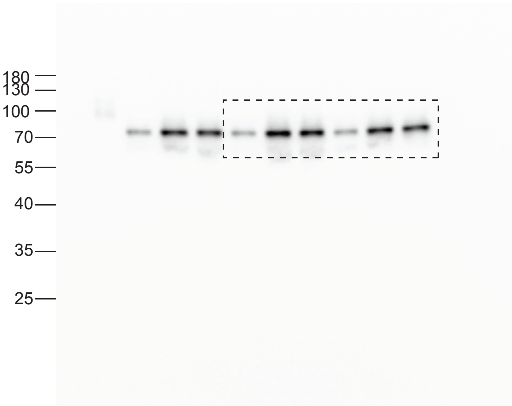

anti-p-p65

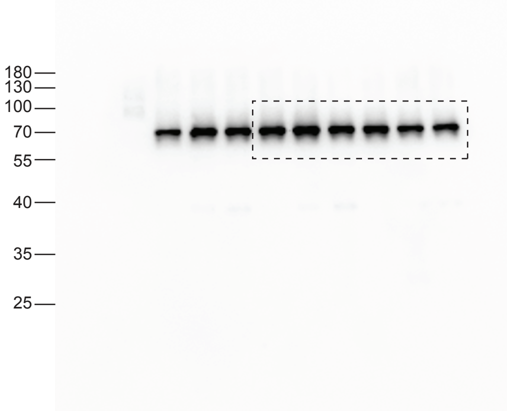

anti-p65

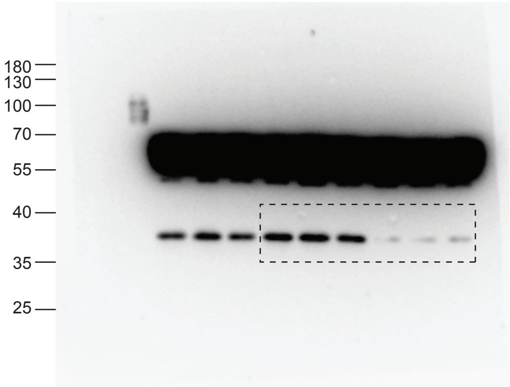

anti-TASL

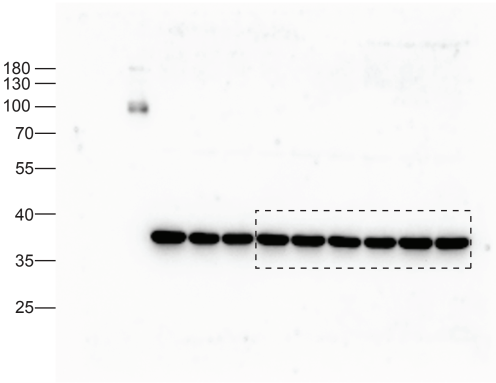

anti-GAPDH

Source Data, Figure 2F

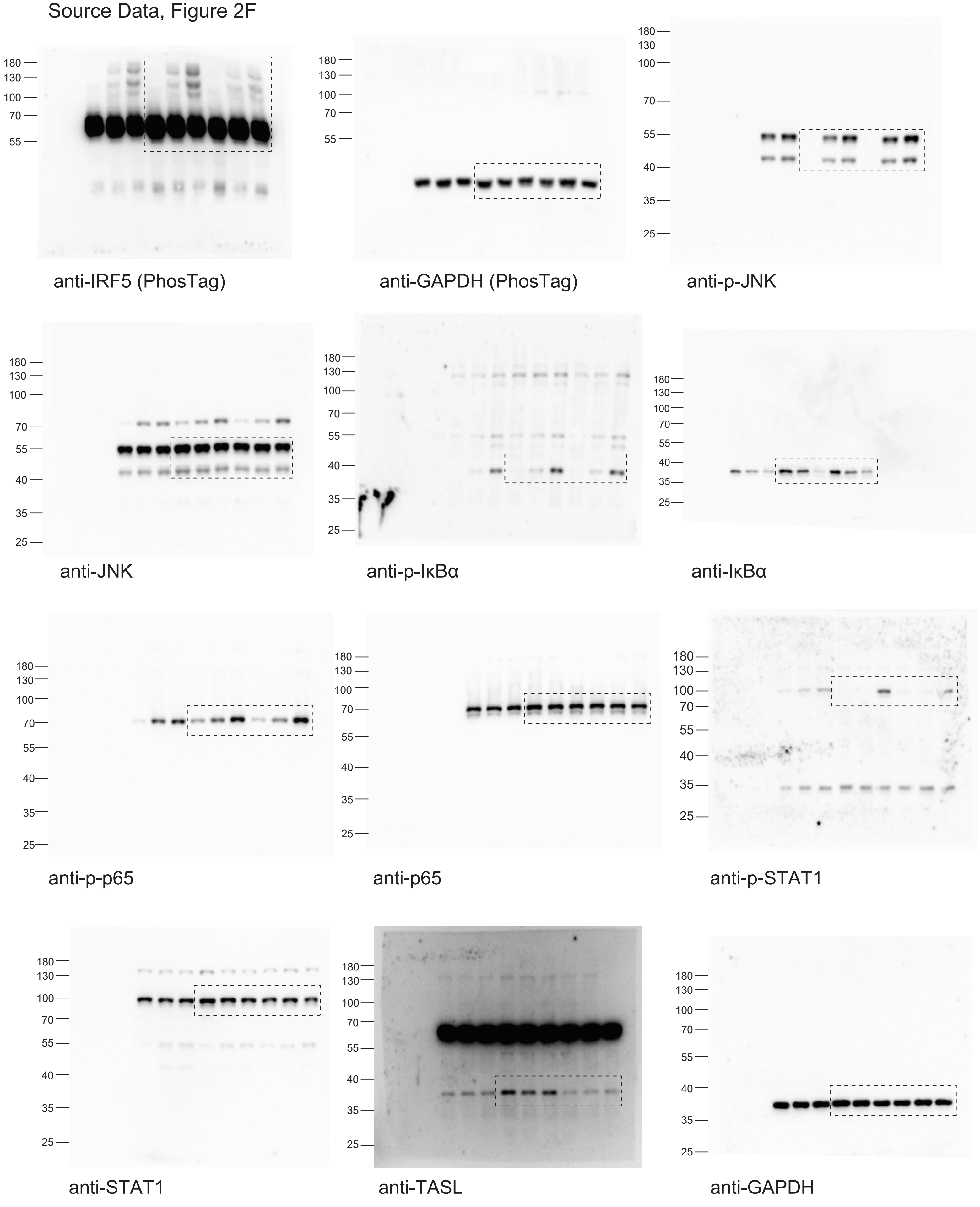

Source data, Figure 3I

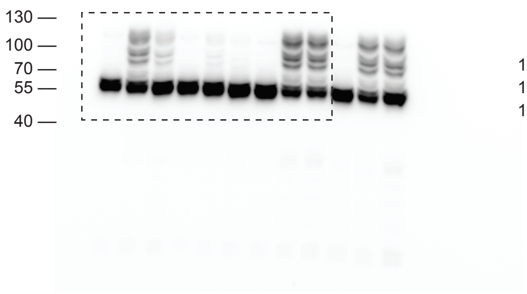

anti-IRF5

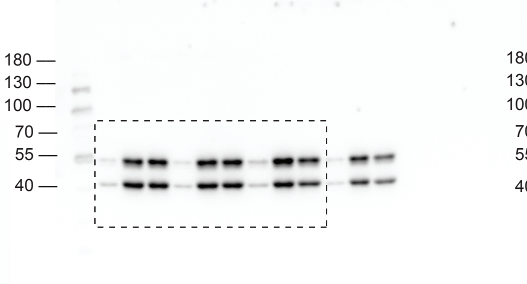

anti-p-JNK

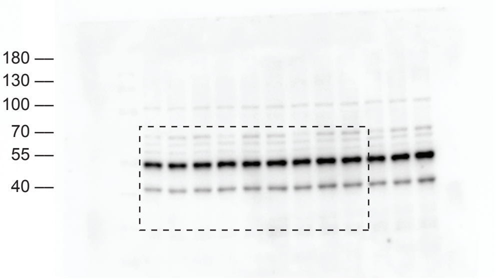

anti-JNK

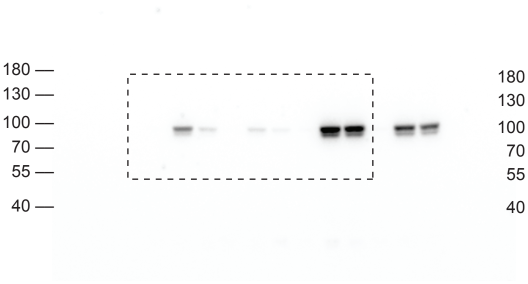

anti-p-STAT1

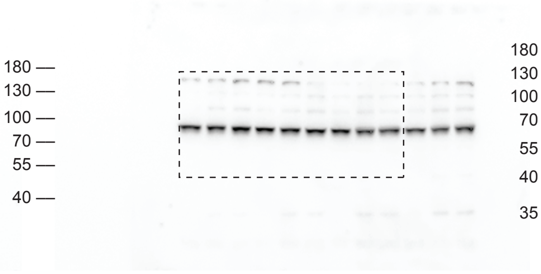

anti-STAT1

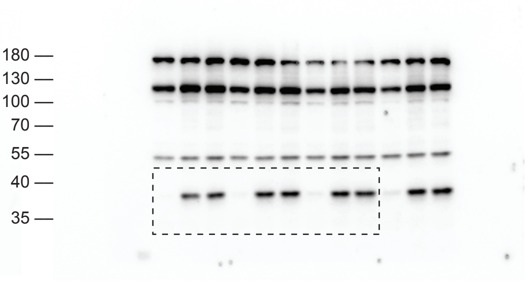

anti-p-IκBα

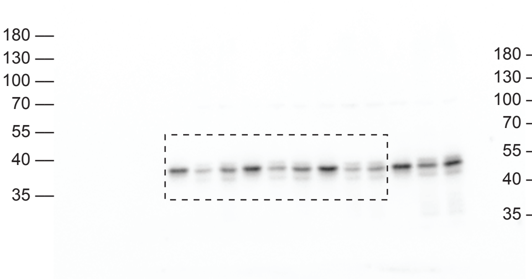

anti-IκBα

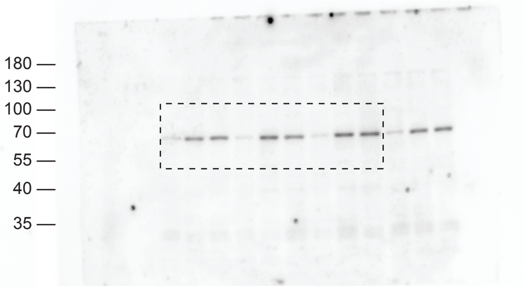

anti-p-p65

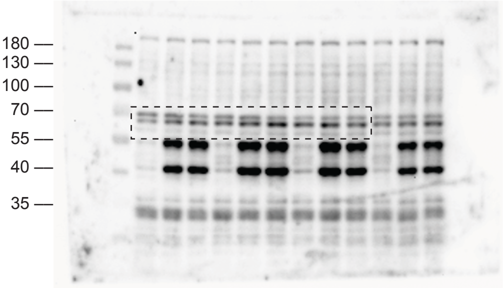

anti-p65

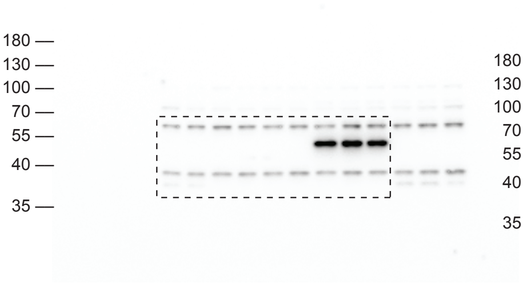

anti-TASL (short exposure)

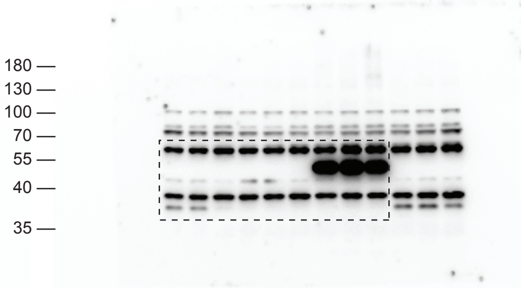

anti-TASL (long exposure)

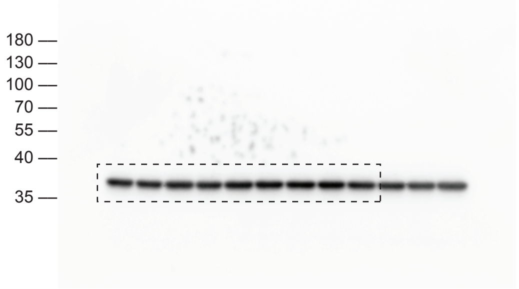

anti-GAPDH

Source Data, Supplementary Figure 2A

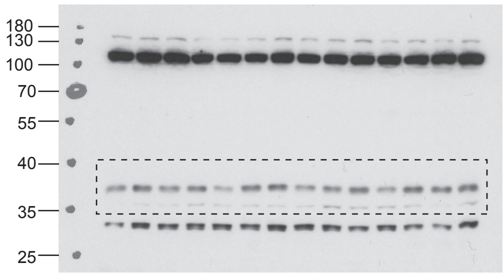

anti-TASL

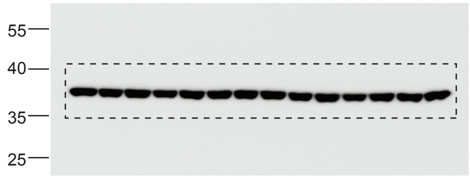

anti-GAPDH

Source Data, Supplementary Figure 4A

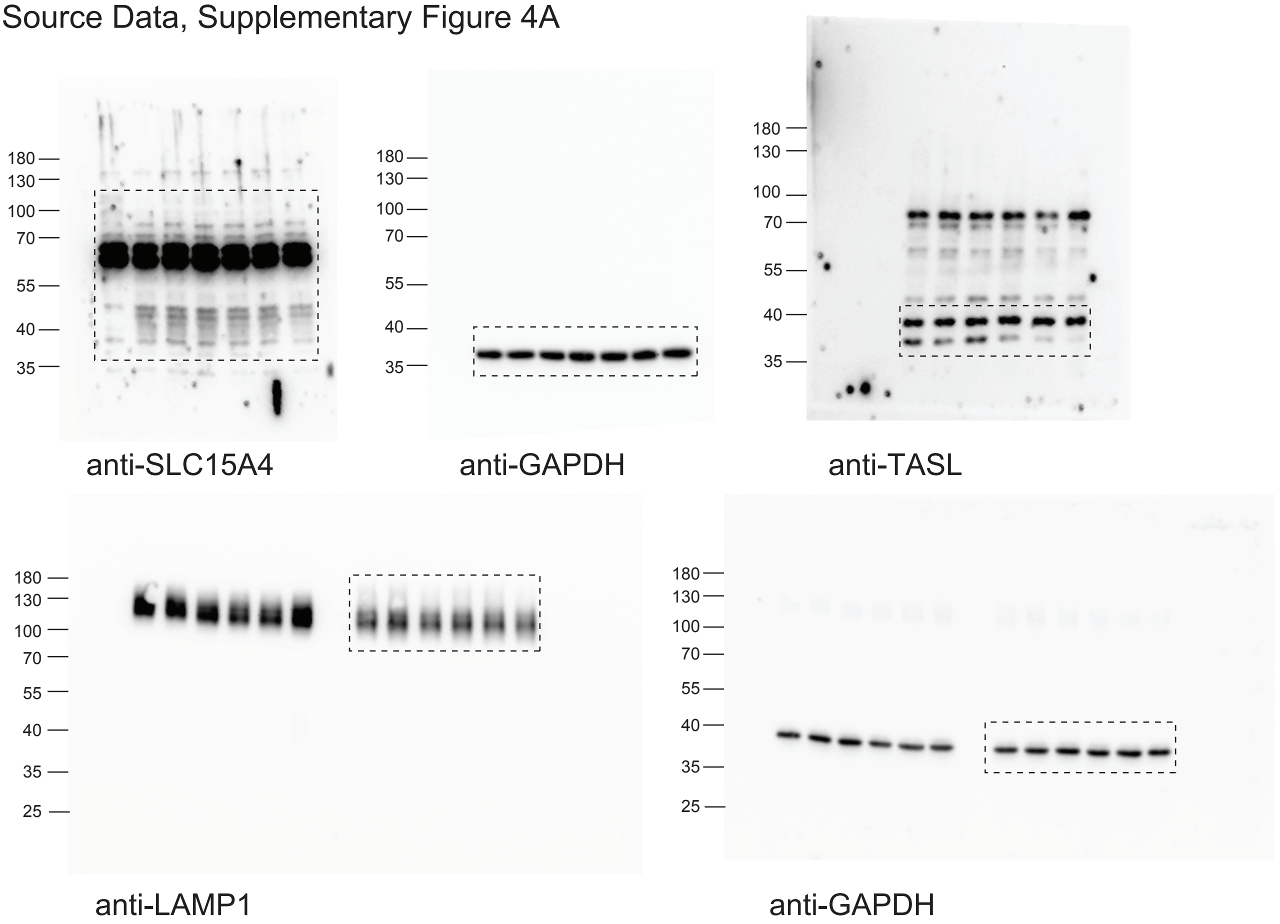

Source Data, Supplementary Figure 4B

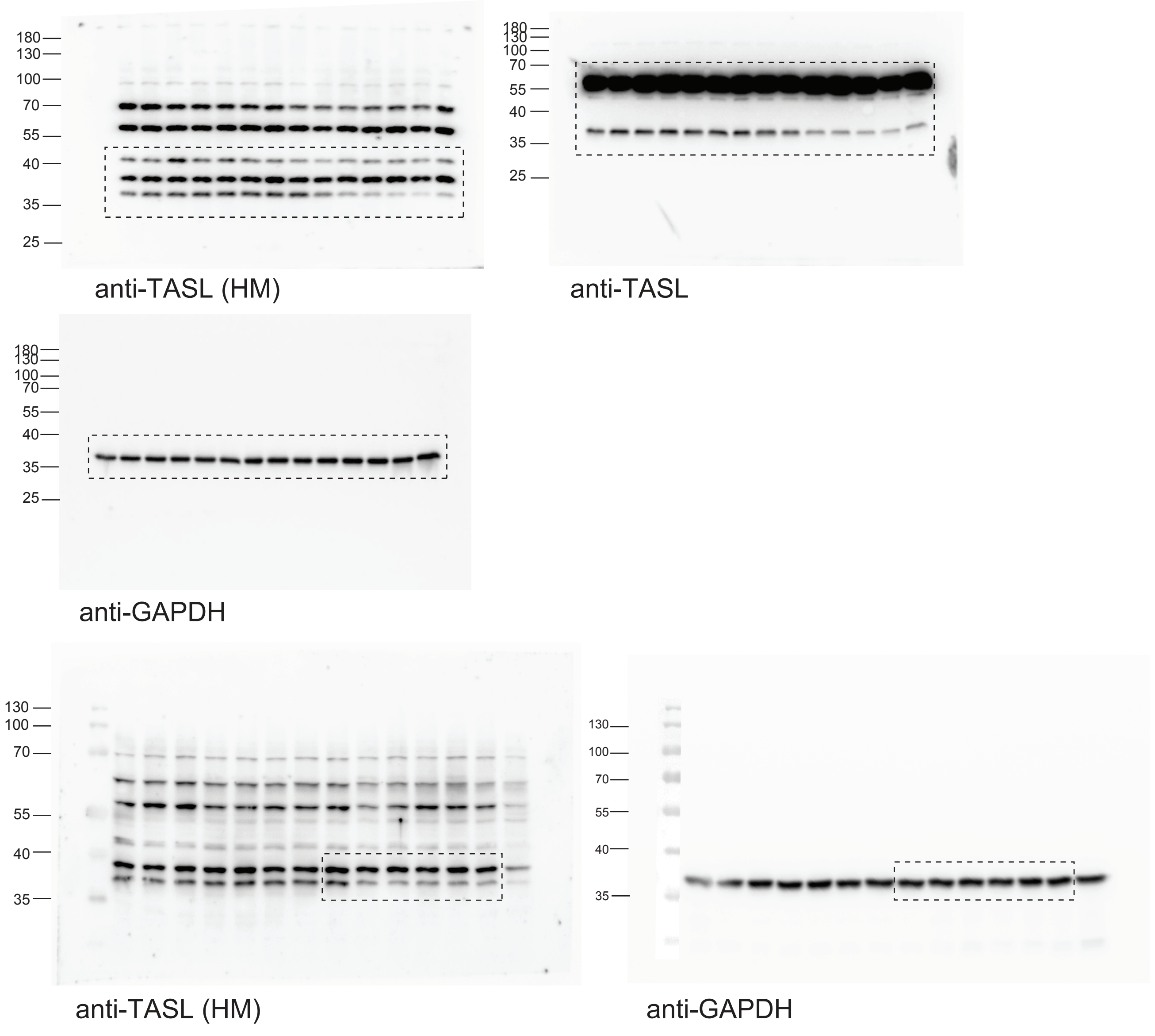

Source Data, Figure S4C

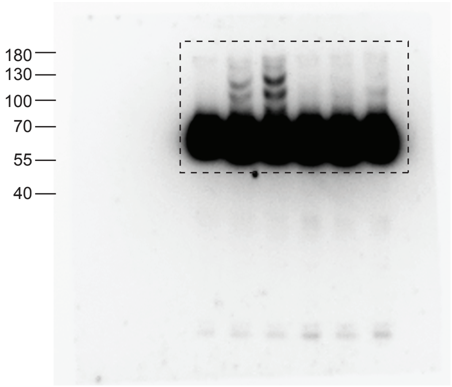

anti-IRF5 (PhosTag)

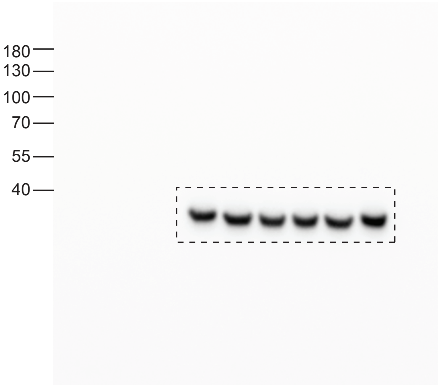

anti-GAPDH (PhosTag)

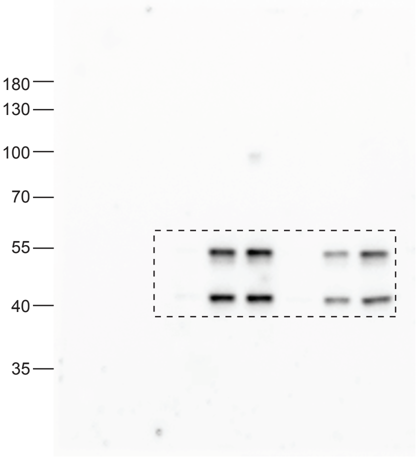

anti-p-JNK

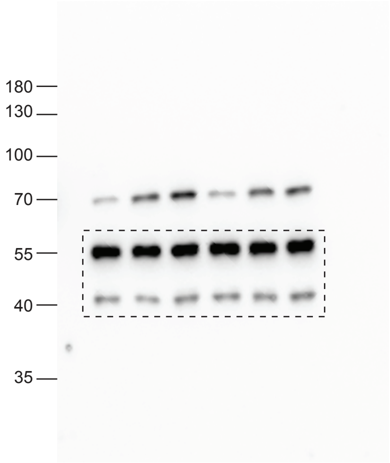

anti-JNK

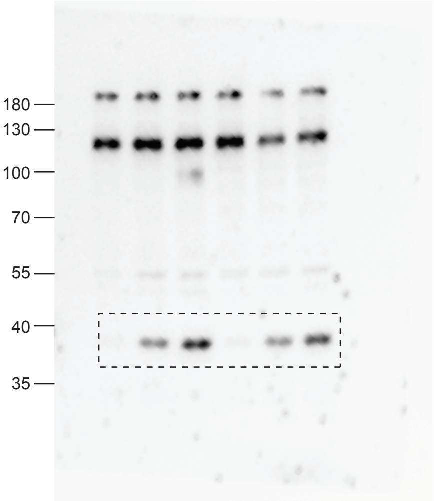

anti-p-IκBα

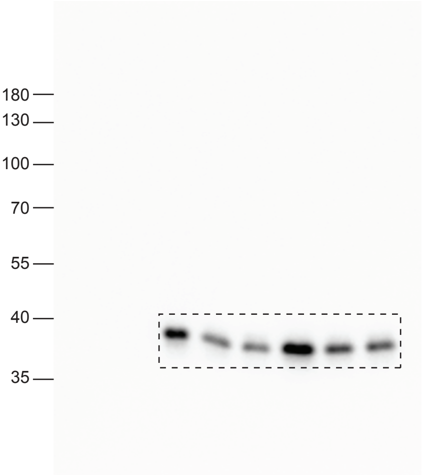

anti-IκBα

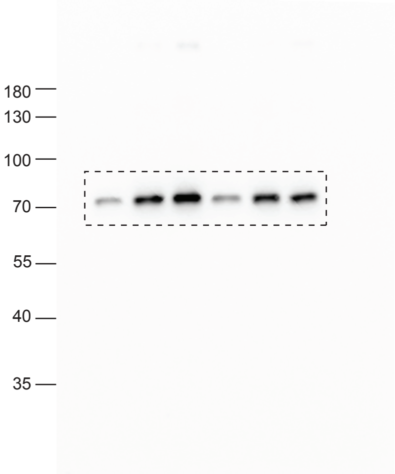

anti-p-p65

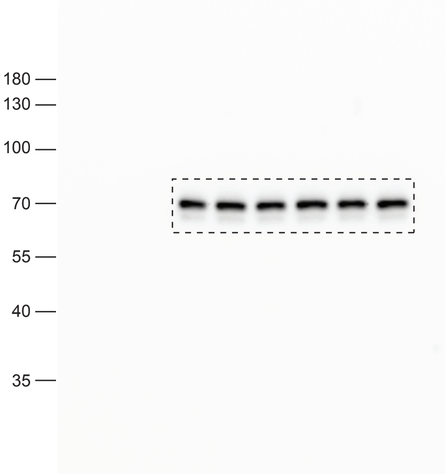

anti-p65

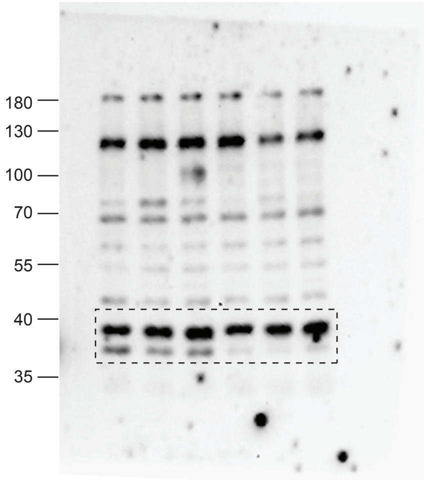

anti-TASL (HM)

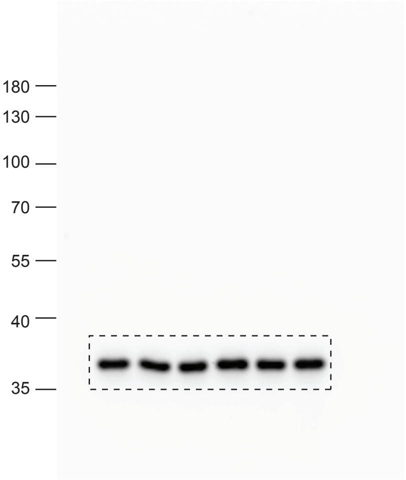

anti-GAPDH

Source Data, Figure S4D

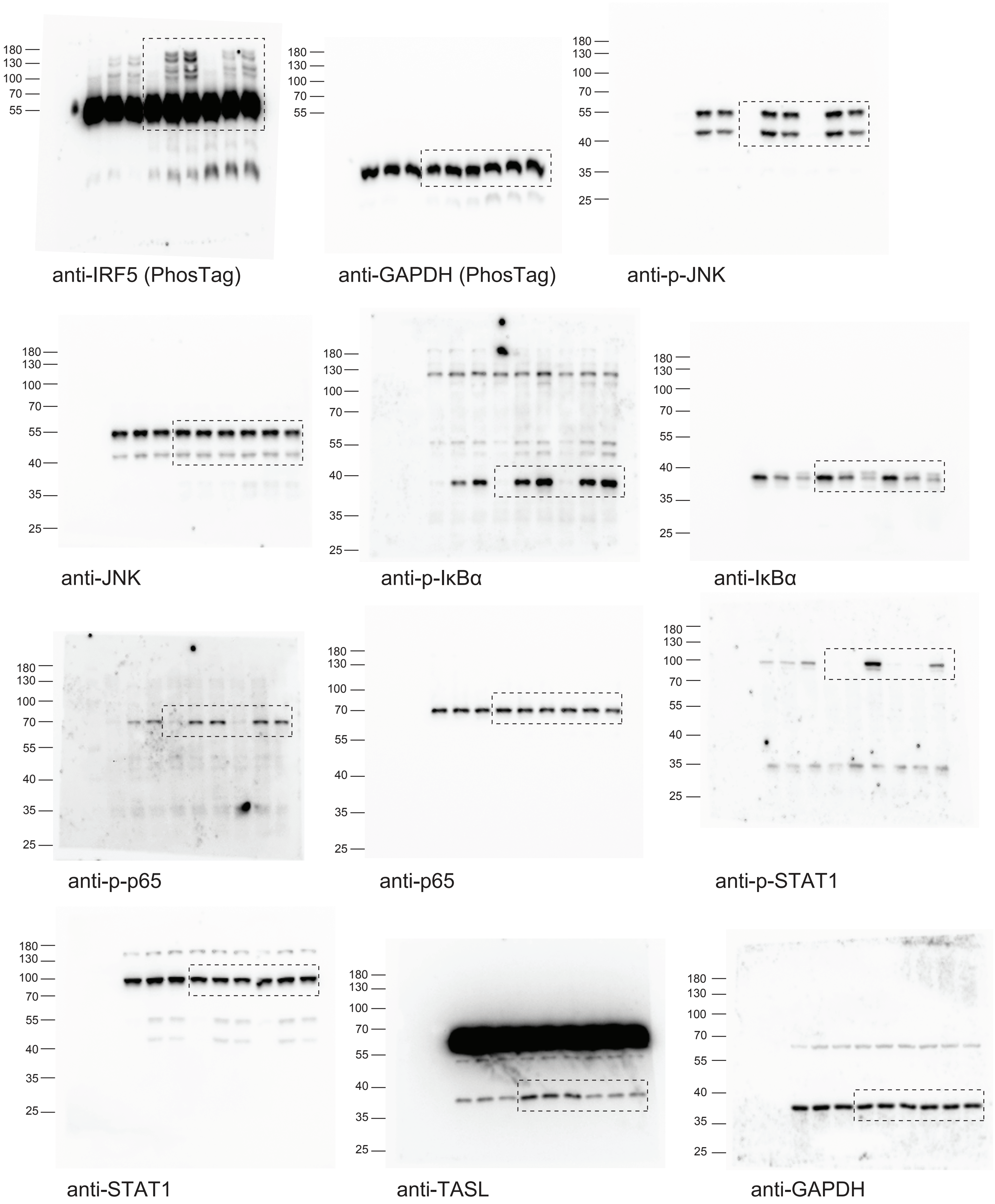

Supplement: Supplementary file 1 — Supplementary Information [file 41467_2023_42070_MOESM1_ESM.pdf]
