# Supplementary material for: A conformation-locking inhibitor of SLC15A4 with TASL proteostatic anti-inflammatory activity
Source: Nat Commun. 2023 Oct 20;14:6626. doi: 10.1038/s41467-023-42070-3 (PMC10589233; doi:10.1038/s41467-023-42070-3)
Supplement: Supplementary file 3 — Reporting Summary [file 41467_2023_42070_MOESM3_ESM.pdf]

nature portfolio

Corresponding author(s): Superti-Furga, Heinz, Rebsamen

Last updated by author(s): Sep 20, 2023

Reporting Summary

Nature Portfolio wishes to improve the reproducibility of the work that we publish. This form provides structure for consistency and transparency in reporting. For further information on Nature Portfolio policies, see our Editorial Policies and the Editorial Policy Checklist.

Statistics

For all statistical analyses, confirm that the following items are present in the figure legend, table legend, main text, or Methods section.

n/a	Confirmed	
☐	☒	The exact sample size (n) for each experimental group/condition, given as a discrete number and unit of measurement
☐	☒	A statement on whether measurements were taken from distinct samples or whether the same sample was measured repeatedly
☐	☒	The statistical test(s) used AND whether they are one- or two-sided<br>*Only common tests should be described solely by name; describe more complex techniques in the Methods section.*
☐	☒	A description of all covariates tested
☐	☒	A description of any assumptions or corrections, such as tests of normality and adjustment for multiple comparisons
☐	☒	A full description of the statistical parameters including central tendency (e.g. means) or other basic estimates (e.g. regression coefficient) AND variation (e.g. standard deviation) or associated estimates of uncertainty (e.g. confidence intervals)
☐	☒	For null hypothesis testing, the test statistic (e.g. F, t, r) with confidence intervals, effect sizes, degrees of freedom and P value noted<br>*Give P values as exact values whenever suitable.*
☒	☐	For Bayesian analysis, information on the choice of priors and Markov chain Monte Carlo settings
☒	☐	For hierarchical and complex designs, identification of the appropriate level for tests and full reporting of outcomes
☒	☐	Estimates of effect sizes (e.g. Cohen's d, Pearson's r), indicating how they were calculated

Our web collection on statistics for biologists contains articles on many of the points above.

Software and code

Policy information about availability of computer code

Data collection	Luciferase/SEAP reporter data: Envision 2104<br>ELISA: Molecular Devices, SoftMax Pro v.7.0; BioTek Gen5 Software v2.0.9<br>Western Blot Detection: Vilber Fusion Solo S7, Vilber EvolutionCapt-v18.12<br>Flow cytometry: BD CellQuest Pro v6.0; BD FACS DIVA v8.0.1<br>Microscopy: Carl Zeiss Ag, Zen 3.6; PerkinElmer Harmony 4.9<br>Structural Modeling: AlphaFold-Multimer v2.1<br>Cryo-EM: AutoEMation 2.0
Data analysis	ELISA and Luciferase/SEAP reporter data: GraphPad Prism v9<br>Flow cytometry: FlowJo v10<br>Microscopy: CellProfiler v4.2.4, R version 4.2.1, ImageJ2 v2.9.0<br>Structural Modeling: PyMol v2.5.0<br>For Cryo-EM data processing: cryoSPARC V3.2, PHENIX1.14-3374, ResMap1.1.4<br>For Cryo-EM atomic model refinement and analysis: Coot0.8.9, PHENIX1.14-3374, MolProbity (part of PHENIX package)<br>For Cryo-EM figure preparation: Pymol 2.1.0, Chimera 1.14, Chimera X 1.25

For manuscripts utilizing custom algorithms or software that are central to the research but not yet described in published literature, software must be made available to editors and reviewers. We strongly encourage code deposition in a community repository (e.g. GitHub). See the Nature Portfolio guidelines for submitting code & software for further information.

Data

Policy information about availability of data

All manuscripts must include a data availability statement. This statement should provide the following information, where applicable:

- Accession codes, unique identifiers, or web links for publicly available datasets
- A description of any restrictions on data availability
- For clinical datasets or third party data, please ensure that the statement adheres to our policy

The 3D cryo-electron microscopy density map and the coordinates of atomic models has been deposited in the Electron Microscopy Data Bank (EMDB) and the Protein Data Bank (PDB) with the following accession codes: EMDB-36754 [https://www.ebi.ac.uk/pdbe/entry/emdb/EMD-36754] and PDB 8JZX [http://doi.org/10.2210/pdb8jzx/pdb] for SLC15A4/C5 complex. The coordinates for previously published SLC15A1 used in Figure S10d were obtained from the PDB database accession code 7PMW [https://doi.org/10.2210/pdb7pmw/pdb]. All other data are available in the manuscript or in the supplementary materials. Noncommercial reagents described in this manuscript are available from MR, LH or GSF under a material transfer agreement. Source data of relevant information are provided as source data files.

Research involving human participants, their data, or biological material

Policy information about studies with human participants or human data. See also policy information about sex, gender (identity/presentation), and sexual orientation and race, ethnicity and racism.

Reporting on sex and gender	N/A as samples from anonymous donors were used.
Reporting on race, ethnicity, or other socially relevant groupings	N/A as samples from anonymous donors were used.
Population characteristics	Anonymous Healthy Donors and SLE patients.
Recruitment	Healthy donors and SLE patients were recruited at the Department of Internal Medicine 3, Division of Rheumatology at the Medical University of Vienna.
Ethics oversight	Ethics Committee of the Medical University of Vienna (EK2071/2020 and EK1075/2021).

Note that full information on the approval of the study protocol must also be provided in the manuscript.

Field-specific reporting

Please select the one below that is the best fit for your research. If you are not sure, read the appropriate sections before making your selection.

☒ Life sciences ☐ Behavioural & social sciences ☐ Ecological, evolutionary & environmental sciences

For a reference copy of the document with all sections, see nature.com/documents/nr-reporting-summary-flat.pdf

Life sciences study design

All studies must disclose on these points even when the disclosure is negative.

Sample size	No statistical method was used to predetermine sample size.
Data exclusions	No data were excluded from the study.
Replication	Reported experiments were repeated at least 2 times with comparable results.
Randomization	The experiments were not randomized.
Blinding	The Investigators were not blinded to allocation during experiments and outcome assessment.

Reporting for specific materials, systems and methods

We require information from authors about some types of materials, experimental systems and methods used in many studies. Here, indicate whether each material, system or method listed is relevant to your study. If you are not sure if a list item applies to your research, read the appropriate section before selecting a response.

Materials & experimental systems

n/a	Involved in the study	
	x	Antibodies
	x	Eukaryotic cell lines
x		Palaeontology and archaeology
x		Animals and other organisms
x		Clinical data
x		Dual use research of concern
x		Plants

Methods

n/a	Involved in the study	
x		ChIP-seq
	x	Flow cytometry
x		MRI-based neuroimaging

Antibodies

Antibodies used

Antibodies used in western blot:

Rabbit anti-TASL (Sigma, Cat.#: HPA001185, Lot: 000030856, Dilution 1:1000), rabbit anti-IRF5 (Abcam, Cat.#: ab181553, Clone: EPR17067, Lot: GR3248905-4, Dilution 1:1000), mouse anti-LAMP1 (Santa Cruz, Cat.#: sc-20011, Clone: H4A3, Lot: H0321, Dilution 1:1000), mouse anti-GAPDH (Santa Cruz, Cat.#: sc-365062, Clone: G-9, Lot: G0121 and I2321, Dilution 1:1000), mouse anti-IκBα (Cell Signaling, Cat.#: 4814, Clone: L35A5, Lot: 17, Dilution 1:1000), rabbit anti-phospho-IκBα Ser32 (Cell Signaling, Cat.#: 2859, Clone: 14D4, Lot: 18, Dilution 1:1000), rabbit anti-SAPK/JNK (Cell Signaling, Cat.#: 9252, Lot: 17, Dilution 1:1000), rabbit anti-phospho-SAPK/JNK Thr183/Tyr185 (Cell Signaling, Cat.#: 4668, Lot: 15, Clone: 81E11, Dilution 1:1000), rabbit anti-STAT1 (Cell Signaling, Cat.#: 14994, Clone: D1K9Y, Lot: 5, Dilution 1:1000), rabbit anti-phospho-STAT1 Tyr701 (Cell Signaling, Cat.#: 7649, Clone: D4A7, Lot: 5, Dilution 1:1000), rabbit anti-NF-κB p65 (Cell Signaling, Cat.#: 8242, Clone: D14E12, Lot: 16, Dilution 1:1000), rabbit anti-phospho-NF-κB p65 Ser536 (Cell Signaling, Cat.#: 3033, Clone: 93H1, Lot: 17, Dilution 1:1000), rabbit anti-HA (Cell Signaling, Cat.#: 3724, Clone: C29F4, , Lot: 10, Dilution 1:1000), rabbit anti-GFP (Cell Signaling, Cat.#: 2956, Clone: D5.1, Lot: 6, Dilution 1:1000). Custom rabbit anti-SLC15A4 antibodies were generated by Genscript (raised against the N-terminus of SLC15A4, Dilution 1:1000, Ref. #23). Custom rabbit anti-TASL antibodies (TASL HM) were produced by Eurogentec (recognizing the C-terminus of TASL, Dilution 1:1000, Ref. #30).

Antibodies used in immunofluorescence:

Rabbit anti-IRF5-Alexa647 (Cell Signaling, Cat.#: 74818, Clone: E7F9W, Lot: 1, Dilution 1:200), rabbit anti-NF-κB p65-Alexa488 (Cell Signaling, Cat.#: 49445, Clone: D14E12, Lot: 3, Dilution 1:200), mouse anti-CD19-PE (BioLegend, Cat.#: 302254, Clone: HIB19, Lot: B325450, Dilution 1:200), mouse anti-V5 (Invitrogen, Cat.#: R960-25, Clone: SV5-Pk1, Lot: 2378586, Dilution 1:1000), goat anti-mouse Alexa-568 (Invitrogen, Cat.#: A11004, Lot: 2090670, Dilution 1:500).

Antibodies used in flow cytometry:

Rabbit anti-V5 (Cell Signaling, Cat.#: 13202, Clone: D3H8Q, Lot: 6, Dilution 1:1000), goat anti-rabbit IgG (H+L), F(ab’) 2 fragment-Alexa647 (Cell Signaling, Cat.#: 4414, Dilution 1:1000).

Validation

Specificity of custom rabbit anti-SLC15A4 (Genscript) and anti-TASL (TASL HM, Eurogentec) have been validated previously (Heinz et al., Ref. #23; Zhang et al., Ref. #30). All other antibodies were bought from commercial vendors and validation for indicated species and applications can be found on the manufacturers website or the provided scientific citations on the same website:

rabbit anti-TASL (Sigma, Cat.#: HPA00118): https://www.sigmaaldrich.com/AT/de/product/sigma/hpa001185

rabbit anti-IRF5 (Abcam, Cat.#: ab181553): https://www.abcam.com/products/primary-antibodies/irf5-antibody-epr17067-ab181553.html

mouse anti-LAMP1 (Santa Cruz, Cat.#: sc-20011): https://www.scbt.com/p/lamp-1-antibody-h4a3

mouse anti-GAPDH (Santa Cruz, Cat.#: sc-365062): https://www.scbt.com/p/gapdh-antibody-g-9

mouse anti-IκBα (Cell Signaling, Cat.#: 4814): https://www.cellsignal.com/products/primary-antibodies/ikba-l35a5-mouse-mab-amino-terminal-antigen/4814

rabbit anti-phospho-IκBα Ser32 (Cell Signaling, Cat.#: 2859): https://www.cellsignal.com/products/primary-antibodies/phospho-ikba-ser32-14d4-rabbit-mab/2859

rabbit anti-SAPK/JNK (Cell Signaling, Cat.#: 9252): https://www.cellsignal.com/products/primary-antibodies/sapk-jnk-antibody/9252

rabbit anti-phospho-SAPK/JNK Thr183/Tyr185 (Cell Signaling, Cat.#: 4668): https://www.cellsignal.com/products/primary-antibodies/phospho-sapk-jnk-thr183-tyr185-81e11-rabbit-mab/4668

rabbit anti-STAT1 (Cell Signaling, Cat.#: 14994): https://www.cellsignal.com/products/primary-antibodies/stat1-d1k9y-rabbit-mab/14994

rabbit anti-phospho-STAT1 Tyr701 (Cell Signaling, Cat.#: 7649): https://www.cellsignal.com/products/primary-antibodies/phospho-

stat1-tyr701-d4a7-rabbit-mab/7649

rabbit anti-NF-κB p65 (Cell Signaling, Cat.#: 8242): https://www.cellsignal.com/products/primary-antibodies/nf-kb-p65-d14e12-xp-rabbit-mab/8242

rabbit anti-phospho-NF-κB p65 Ser536 (Cell Signaling, Cat.#: 3033): https://www.cellsignal.com/products/primary-antibodies/phospho-nf-kb-p65-ser536-93h1-rabbit-mab/3033

rabbit anti-HA (Cell Signaling, Cat.#: 3724): https://www.cellsignal.com/products/primary-antibodies/ha-tag-c29f4-rabbit-mab/3724

rabbit anti-GFP (Cell Signaling, Cat.#: 2956): https://www.cellsignal.com/products/primary-antibodies/gfp-d5-1-rabbit-mab/2956

anti-IRF5-Alexa647 (Cell Signaling, Cat.#: 74818): https://www.cellsignal.com/products/antibody-conjugates/irf-5-e7f9w-rabbit-mab-alexa-fluor-647-conjugate/74818

rabbit anti-NF-κB p65-Alexa488 (Cell Signaling, Cat.#: 49445): https://www.cellsignal.com/products/antibody-conjugates/nf-kb-p65-d14e12-xp-rabbit-mab-alexa-fluor-488-conjugate/49445

mouse anti-CD19-PE (BioLegend, Cat.#: 302254): https://www.biolegend.com/de-at/products/pe-anti-human-cd19-antibody-719?GroupID=BLG10095

mouse anti-V5 (Invitrogen, Cat.#: R960-25): https://www.thermofisher.com/antibody/product/V5-Tag-Antibody-clone-SV5-Pk1-Monoclonal/R960-25

goat anti-mouse Alexa-568 (Invitrogen, Cat.#: A11004): https://www.thermofisher.com/antibody/product/Goat-anti-Mouse-IgG-H-L-Cross-Adsorbed-Secondary-Antibody-Polyclonal/A-11004

rabbit anti-V5 (Cell Signaling, Cat.#: 13202): https://www.cellsignal.com/products/primary-antibodies/v5-tag-d3h8q-rabbit-mab/13202

goat anti-rabbit IgG (H+L), F(ab') 2 fragment-Alexa647 (Cell Signaling, Cat.#: 4414): https://www.cellsignal.com/products/secondary-antibodies/anti-rabbit-igg-h-l-f-ab-2-fragment-alexa-fluor-647-conjugate/4414

Eukaryotic cell lines

Policy information about cell lines and Sex and Gender in Research

Cell line source(s)	HEK293T cells (Cat. #: CRL-3216) and THP1 cells (Cat. #: TIB-202) were purchased from ATCC, THP1 DUAL reporter cell lines Cat. #: dhpd-nfis) from Invivogen. CAL-1 cells were kindly provided by Prof. Takahiro Maeda.
Authentication	HEK293T and THP1 cells were authenticated by STR profiling. CAL-1 (obtained directly from Prof. Maeda) and THP1 DUAL cells (obtained from Invivogen) were not authenticated.
Mycoplasma contamination	Cell lines used were tested negatively for mycoplasma contamination.
Commonly misidentified lines (See ICLAC register)	None of commonly misidentified cell lines were used in this study.

Flow Cytometry

Plots

Confirm that:

- [x] The axis labels state the marker and fluorochrome used (e.g. CD4-FITC).
- [x] The axis scales are clearly visible. Include numbers along axes only for bottom left plot of group (a 'group' is an analysis of identical markers).
- [ ] All plots are contour plots with outliers or pseudocolor plots.
- [ ] A numerical value for number of cells or percentage (with statistics) is provided.

Methodology

Sample preparation	Hek293T cells transfected with TASL-V5 plasmid were fixed and permeabilized with IC fixation and permeabilization buffer (Invitrogen) before staining for V5-tag and detected with deep red Alexa Fluor 647 secondary antibody to allow for selection in the presence of an mCherry reporter. Cells were measured either immediately after staining or in the case of live cells immediately after harvesting and washing with PBS
Instrument	BD Biosciences FACSCalibur; BD LSR Fortessa II

Software	Acquisition: BD CellQuest Pro v6.0; BD FACS DIVA v8.0.1; analysis: FlowJo v10
Cell population abundance	No sorting experiment was performed.
Gating strategy	Fixed cells were gated based on 647 signal and live cells were gated based on FSC/SSC and mCherry signal when applicable.

☒ Tick this box to confirm that a figure exemplifying the gating strategy is provided in the Supplementary Information.
